# IndoHotelABSA: A large-scale aspect-based sentiment analysis dataset of Indonesian hotel reviews for hospitality CRM

**DOI:** 10.1016/j.dib.2026.113209

**Published:** 2026-09-03

**Authors:** Xaverius Sika, Eko Sediyono, Irwan Sembiring, Teguh Wahyono, Eko Arip Winanto

**Affiliations:** a Department of Information System, Universitas Dinamika Bangsa (UNAMA), Jambi, Indonesia; b Faculty of Information Technology, Satya Wacana Christian University (UKSW), Salatiga, Indonesia; c Department of Computer Engineering, Universitas Dinamika Bangsa (UNAMA), Jambi, Indonesia

**Keywords:** Aspect-based sentiment analysis, Indonesian NLP, Low-resource, Hotel reviews, Benchmark dataset, Customer relationship management

## Abstract

IndoHotelABSA is a large-scale dataset for aspect-based sentiment analysis (ABSA) of Indonesian hotel reviews. It comprises 14,988 unique, deduplicated Indonesian-language reviews collected on 10 July 2026 through the official Google Places API from 3,075 establishments across 55 cities spanning all six major regions of Indonesia. A class-balanced sample of 3,000 reviews was annotated for seven CRM-meaningful aspect categories (Location, Cleanliness, Service/Staff, Room & Facilities, Price/Value, Food & Beverage, and Public Facilities), each with three-way polarity (positive, negative, neutral), using a two-tier protocol: all 3,000 reviews received large-language-model (LLM) draft labels, forming the *silver* tier, and a 500-review subset was checked and corrected independently by three human annotators and adjudicated by majority vote, forming the *gold* tier. The gold subset reaches almost-perfect agreement (Fleiss’ κ=0.894) and yields 1,506 gold aspect annotations. The release explicitly distinguishes the 2,480 unverified silver training records from the 500 human-verified gold records and provides fixed train/validation/test splits. The dataset fills the absence of a public, quality-controlled Indonesian hotel ABSA benchmark and can be reused for low-resource NLP, LLM-annotation research, and hospitality customer relationship management (CRM) analytics. All files are provided as UTF-8 JSON Lines with a datasheet and annotation guidelines.

Specifications Table

**Table:** Specifications table for the IndoHotelABSA dataset.

Subject	Computer Science, Artificial Intelligence, Natural Language Processing.
Specific subject area	Aspect-based sentiment analysis (ABSA) of Indonesian hotel reviews for hospitality CRM.
Type of data	Text (reviews), annotations (aspect–polarity labels), tables and figures (summary statistics).
Data collection	Indonesian-language hotel reviews were retrieved on 10 July 2026 through the official Google Places API (Text Search and Place Details endpoints, language=id) for 55 cities across all six major Indonesian regions. Each city was queried with eight complementary Indonesian search expressions, and establishments were merged and de-duplicated by place identifier at collection time, giving 3,075 establishments. Reviews were deduplicated on a normalised, lower-cased 200-character key and filtered to 20–2,000 characters. A class-balanced sample of 3,000 reviews was drafted by an LLM (silver tier). A 500-review subset was verified and corrected by three annotators and adjudicated by majority vote (gold tier).
Data format	Raw and analysed. UTF-8 JSON Lines (.jsonl) and Markdown documentation.
Description of data collection	Secondary, user-generated content. Only review text, star rating, hotel name, and city were retained. Reviewer-identifying *metadata fields* (reviewer name, profile and photo URLs, author attribution, timestamp) were never requested from the API and were not stored, and place identifiers were used transiently for de-duplication and were likewise not stored. A separate deterministic pass over the review text replaced telephone numbers, e-mail addresses, and URLs with typed placeholders (Section 4.1).
Data source location	Reviews of hotels located across 55 cities in Indonesia (Sumatra, Java, Bali & Nusa Tenggara, Kalimantan, Sulawesi, Maluku & Papua). Curated at Universitas Dinamika Bangsa (UNAMA), Jambi, Indonesia.
Data accessibility	Dataset: Mendeley Data, DOI: 10.17632/9vhzg5wkf9, which always resolves to the current version. This article describes version 2, DOI: 10.17632/9vhzg5wkf9.2. *Licence is split by component*: the annotations, aspect schema, annotation guidelines, split definitions, datasheet, summary statistics, and all code are released under CC BY 4.0. The review text and hotel names are third-party, user-generated content served through Google Places, redistributed for non-commercial academic research only with attribution and *not* relicensed (see LICENSE_DATA.md and the Ethics Statement). Code: GitHub https://github.com/robotumbel/IndoHotelABSA (MIT), archived and citable on Zenodo, DOI: 10.5281/zenodo.21990359.
Related research article	X. Sika, E. Sediyono, I. Sembiring, T. Wahyono, E.A. Winanto, “IndoHotelABSA: An Explainable Aspect-Based Sentiment Analysis Framework and Benchmark Dataset for Indonesian Hotel Reviews in Hospitality CRM,” *Natural Language Processing Journal* (submitted) [1].

## 1 Value of the Data

• It is, to our knowledge, the first large-scale, publicly available ABSA resource for *Indonesian hotel reviews*, pairing a human-verified 500-review gold benchmark with a 2,480-review silver training tier and addressing a clear low-resource gap. The closest prior resource (HoASA in IndoNLU [2]) is an order of magnitude smaller, single-platform, and uses a non-CRM aspect inventory.
• It enables reproducible benchmarking and comparison of classical, encoder-based, and large-language-model ABSA methods for Indonesian, using fixed, documented train/validation/test splits. Evaluation should be reported on the gold splits.
• Its seven CRM-aligned aspect categories make it directly reusable for hospitality analytics, aspect dashboards, and customer relationship management research, and adaptable to other service domains.
• The explicit silver/gold labelling and the fully disclosed LLM-assisted protocol, including the verbatim prompt (Appendix Appendix A) and the three annotators’ raw label files, support research on LLM-based annotation, semi-supervised learning, label-noise, and annotation-cost trade-offs.
• Nationwide coverage (3,075 establishments, 55 cities, six major regions) supports studies of regional variation, informal/code-mixed Indonesian, and cross-domain or cross-lingual transfer.

## 2 Background

Online guest reviews have become the primary channel through which hotels receive continuous, unsolicited feedback, and for hospitality customer relationship management (CRM) they are the richest available source of voice-of-customer information [3]. Turning this unstructured text into operational insight, however, is difficult, because a single review typically mixes praise and complaint across several facets of the guest experience. Document-level sentiment analysis, which assigns a single polarity to an entire review [4], [5], necessarily collapses these distinct signals. Aspect-based sentiment analysis (ABSA) was introduced to recover this structure by decomposing a text into (aspect, polarity) pairs [6], [7], and for hospitality CRM the aspect dimension is the actionable unit: a manager needs to know *which* operational domain — cleanliness, service, or pricing — is driving dissatisfaction in order to intervene.

Methodologically, sentiment analysis has advanced from lexicon- and feature-based methods to deep-learning approaches [5], [8], [9], and ABSA in particular has been reshaped by the Transformer architecture [10] and pre-trained language models such as BERT [11], with cross-lingual encoders like XLM-RoBERTa [12] extending coverage to many languages. The remaining open challenges are catalogued in recent surveys [7], [13]. This progress is, however, overwhelmingly English-centric, and fine-grained ABSA has only recently reached other low-resource languages, for example Bengali aspect sentiment triplet extraction [14], Arabic dataset creation with explainability [15], and large language model studies for Swahili and code-switched Arabic [16], [17]. A recurring prerequisite across these efforts is a well-documented, quality-controlled dataset in the target language.

For Indonesian, one of the most widely spoken languages in the world and the dominant language of a fast-growing Southeast Asian tourism market, NLP resources have expanded substantially with benchmarks and pre-trained models such as IndoNLU [2], IndoLEM [18], IndoBERTweet [19], and IndoNLG [20], and with broader consolidation efforts including NusaCrowd [21] and NusaX [22]. Applied domains such as disaster-related named-entity recognition are also emerging [23]. For aspect-based sentiment specifically, however, the only closely related Indonesian hotel resource is HoASA within IndoNLU [2], which is drawn from a single platform, is limited in scale, and uses a platform-specific aspect inventory not aligned with CRM decision dimensions. To our knowledge, no large-scale, nationwide, CRM-aligned ABSA benchmark for Indonesian hotel reviews with an independently verified gold tier existed prior to this work.

IndoHotelABSA closes this gap. It is documented here following the *Datasheets for Datasets* framework [24]. The present article describes the dataset and its construction, while its use to benchmark classical, encoder-based, and large-language-model methods and to build an explainable CRM framework is reported in the companion research article [1].

## 3 Data Description

The dataset is distributed as UTF-8 JSON Lines files (one JSON object per line) together with Markdown documentation (Table 1). The full corpus (dataset_merged.jsonl) contains 14,988 unlabelled reviews, and the labelled files provide silver (LLM-drafted) and gold (human-verified) aspect–polarity annotations.

**Table 1:** Files in the IndoHotelABSA release.

File	Content	Records
dataset_merged.jsonl	Full unlabelled corpus (text, rating, hotel, city)	14,988
train_silver.jsonl	Silver (LLM-drafted, unverified) training labels	2,480
val_gold.jsonl	Gold (human-verified) validation labels	100
test_gold.jsonl	Gold (human-verified) test labels	400
gold_final.jsonl	Full adjudicated gold set ( = validation ∪ test)	500
excluded_empty_labels.jsonl	Sampled reviews with an empty aspect set (Section 4.4)	20
annotator_{1,2,3}_verified.jsonl	Each annotator’s raw, unadjudicated gold labels	500 each
adjudication_record.jsonl	Per-cell votes, adjudicated label, disagreement flag	3,500 cells
annotation_prefilled_gold500.jsonl	LLM draft shown to annotators, gold subset	500
gold_subset_ids.txt	Identifiers of the 500 gold reviews	500
sample_to_corpus_ids.tsv	Maps labelled review_id to corpus review_id	3,000
annotator_{1,2,3}_blind.jsonl	Blinded re-annotation of a 100-review sub-sample	100 each
blind_subset_ids.txt	Identifiers of the blinded sub-sample	100
blind_kappa_report.md	Blinded vs. verification comparison	–
README.md	Field schema, splits, licence, citation	–
annotation_guidelines.md	Aspect schema, decision rules, verbatim LLM prompt	–
datasheet.md	Datasheet for Datasets (provenance, uses, limits)	–
LICENSE_DATA.md	Licence split, attribution, takedown procedure	–
verify_release.py	Reproduces the counts and overlaps in Table 4	–
scrub_pii.py	Deterministic in-text PII scrubbing pass	–
remove_records.py	Applies a takedown request consistently across all files	–
compute_blind_kappa.py	Reproduces the blinded-round comparison	–
make_corpus_figures.py	Regenerates Figure 1 from the corpus	–
scrape_reviews.py*et al.*	Pipeline scripts named in Section 4: collection, merge, sampling, LLM pre-annotation, adjudication, and agreement	–
verification_report.txt	Saved output of verify_release.py	–
requirements.txt	Python version and pinned dependency versions	–

*Record schema* Each labelled record is a JSON object: {review_id, corpus_review_id, hotel, city, rating, text, labels:[{aspect, polarity}], verified, gold}, where rating∈{1,…,5}. The labels field lists the mentioned aspects with their polarity, and an aspect absent from it is treated as n/a (not mentioned). The two boolean fields make the tier of every record explicit: gold records carry verified: true, gold: true, silver records carry verified: false, gold: false.

Two identifier spaces are in use, and users must not confuse them. Records in the unlabelled corpus are numbered in collection order, whereas the annotation sample was renumbered 1–3,000 when it was drawn, so the same integer denotes different reviews in the two families of files. Every labelled record therefore also carries corpus_review_id, the identifier of the same review in dataset_merged.jsonl, and the complete correspondence is released as sample_to_corpus_ids.tsv. Joins between the labelled files and the corpus must use corpus_review_id. Joining on review_id returns silently mismatched records. The mapping is exact and complete: all 3,000 sampled reviews resolve to a unique corpus record, which verify_release.py checks by comparing the linked texts. The seven aspect categories are stored in Indonesian, *Lokasi* (Location), *Kebersihan* (Cleanliness), *Pelayanan* (Service/Staff), *Kamar & Fasilitas* (Room & Facilities), *Harga* (Price/Value), *Makanan & Minuman* (Food & Beverage), and *Fasilitas Pendukung* (Public Facilities), and polarity is one of positif/negatif/netral.

*Sample records* Table 2 shows representative gold-annotated reviews (quoted verbatim, with English glosses). A single review often mixes polarities across several aspects (Sample 1 carries positive, negative, and neutral aspects at once), and sentiment is frequently expressed through informal, code-mixed language.

**Table 2:** Sample gold-annotated reviews from IndoHotelABSA (verbatim, with English glosses). Polarity: + positive, - negative, 0 neutral.

#	Review (Indonesian, verbatim) / English gloss	Gold annotations
1	*“Harga terlalu tinggi, tapi kamar cukup nyaman, makanan enak, wifi tersedia walau hanya pakai hotspot seluler, petugas sangat ramah...”*“The price is too high, but the room is quite comfortable, the food is good, wifi is available though only via mobile hotspot, the staff are very friendly...”	Price/Value: - , Room & Facilities: + , Food & Beverage: + , Service/Staff: + , Public Facilities:0
2	*“AC gak dingin, kamar mandi bocor gak tau air apaan, sarapan cuma roti ama teh manis”*“The AC isn’t cold, the bathroom leaks some unknown water, breakfast is just bread and sweet tea”	Room & Facilities: - , Food & Beverage: -
3	*“Penginapan dengan pelayanan yang ramah 24 jam, harga bersahabat, kamar nyaman, ac normal...kamar mandi bersih, disediakan handuk dan sandal.”*“A guesthouse with friendly 24-hour service, affordable price, comfortable room, working AC...a clean bathroom, towels and slippers provided.”	Cleanliness: + , Service/Staff: + , Room & Facilities: + , Price/Value: +

**Fig. 1:**
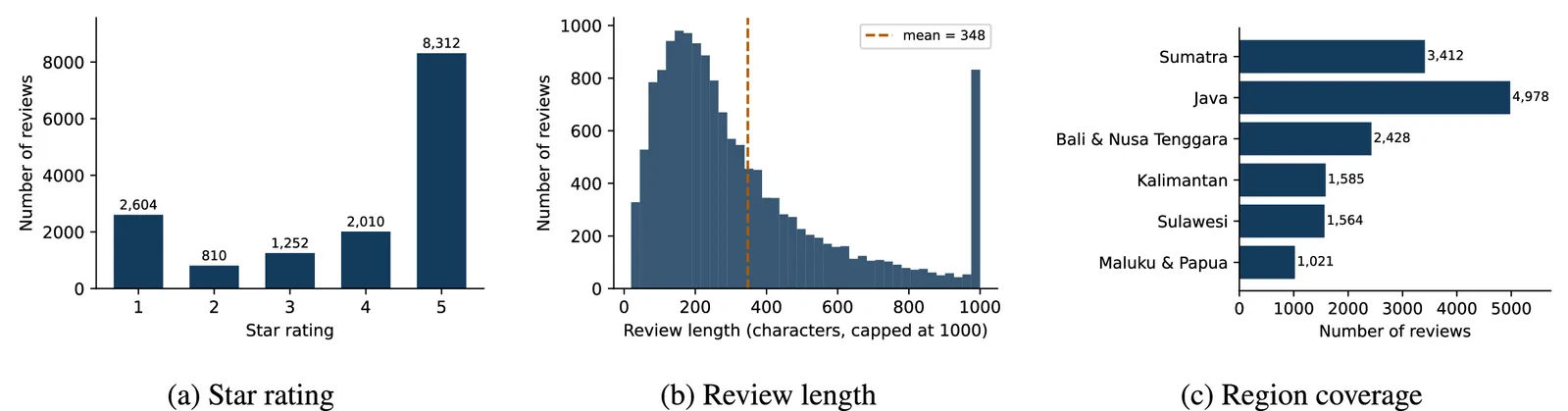
Descriptive statistics of the 14,988-review IndoHotelABSA corpus.

*Corpus statistics* The corpus spans all six major Indonesian regions: Java (4,978 reviews), Sumatra (3,412), Bali & Nusa Tenggara (2,428), Kalimantan (1,585), Sulawesi (1,564), and Maluku & Papua (1,021). Reviews have a mean length of 348 characters (median 250). The star-rating distribution is positively skewed, with 2,604 reviews at one star, 810 at two, 1,252 at three, 2,010 at four, and 8,312 at five. This motivated the class-balanced sampling used for annotation. Figure 1 summarises these distributions.

*Annotation content and quality* The adjudicated gold set (500 reviews) contains 1,506 aspect annotations (727 negative, 710 positive, 69 neutral). The silver tier contains 7,338 aspect annotations (3,258 negative, 3,746 positive, 334 neutral). Over the 500×7=3,500 (review, aspect) cells of the gold subset, all three annotators agreed on 3,170 cells (90.6%). Overall inter-annotator agreement is Fleiss’ κ=0.894 (“almost perfect”) [25]. Pairwise Cohen’s κ [26] ranges from 0.846 to 0.987 (raw agreement 90.9–99.3%), and 92.1% of LLM-drafted cells were retained unchanged after human verification (275 cells, 7.9%, were corrected). Table 3 reports per-aspect κ. As explained in Section 4.4, annotators worked from a visible LLM draft. To establish what that protocol does to these figures, a 100-review sub-sample was re-annotated blind. The comparison is reported in Section 4.4 and shows that agreement is not inflated by the shared draft, but that the gold aspect inventory is conservative.

**Table 3:** Per-aspect Fleiss’ κ over the 500-review gold subset. Annotators verified a visible LLM draft, see Section 4.4.

Aspect	Fleiss’ κ
Location	0.811
Cleanliness	0.872
Service/Staff	0.906
Room & Facilities	0.892
Price/Value	0.905
Food & Beverage	0.922
Public Facilities	0.859
Overall	0.894

*Splits, inclusion, and overlap* The release provides fixed splits: a 2,480-review silver training set, a 100-review gold validation set, and a 400-review gold test set. Of the 3,000 sampled reviews, 500 form the gold subset (released as 100 validation and 400 test) and 2,500 form the silver pool. Twenty of the latter received an empty aspect set from the model, mention none of the seven schema aspects, and were excluded from the silver training file (Section 4.4), giving 2,480 and a released total of 2,480+100+400=2,980. The 20 excluded records are released separately, so that 2,980+20=3,000 is verifiable from the released files alone. Table 4 documents inclusion and overlap among all labelled files. In particular, gold_final.jsonl is exactly the union of val_gold.jsonl and test_gold.jsonl and is provided only as a convenience file, and it must therefore not be used for training when evaluating on test_gold.jsonl. train_silver.jsonl shares no review_id with any gold file. Users evaluating models should report on the gold labels. The silver tier supplies training supervision and is unverified. The script verify_release.py recomputes every row of Table 4 from the released files.

**Table 4:** Inclusion and overlap among the released record sets.

Set	Records	Relationship
dataset_merged.jsonl	14,988	Full corpus, superset of all sets below
Annotation sample	3,000	Drawn from dataset_merged.jsonl
gold_final.jsonl	500	Adjudicated gold set
val_gold.jsonl	100	Subset of gold_final.jsonl
test_gold.jsonl	400	Subset of gold_final.jsonl, disjoint from validation
train_silver.jsonl	2,480	Disjoint from gold_final.jsonl
excluded_empty_labels.jsonl	20	Disjoint from all labelled files

## 4 Experimental Design, Materials and Methods

**Fig. 2:**
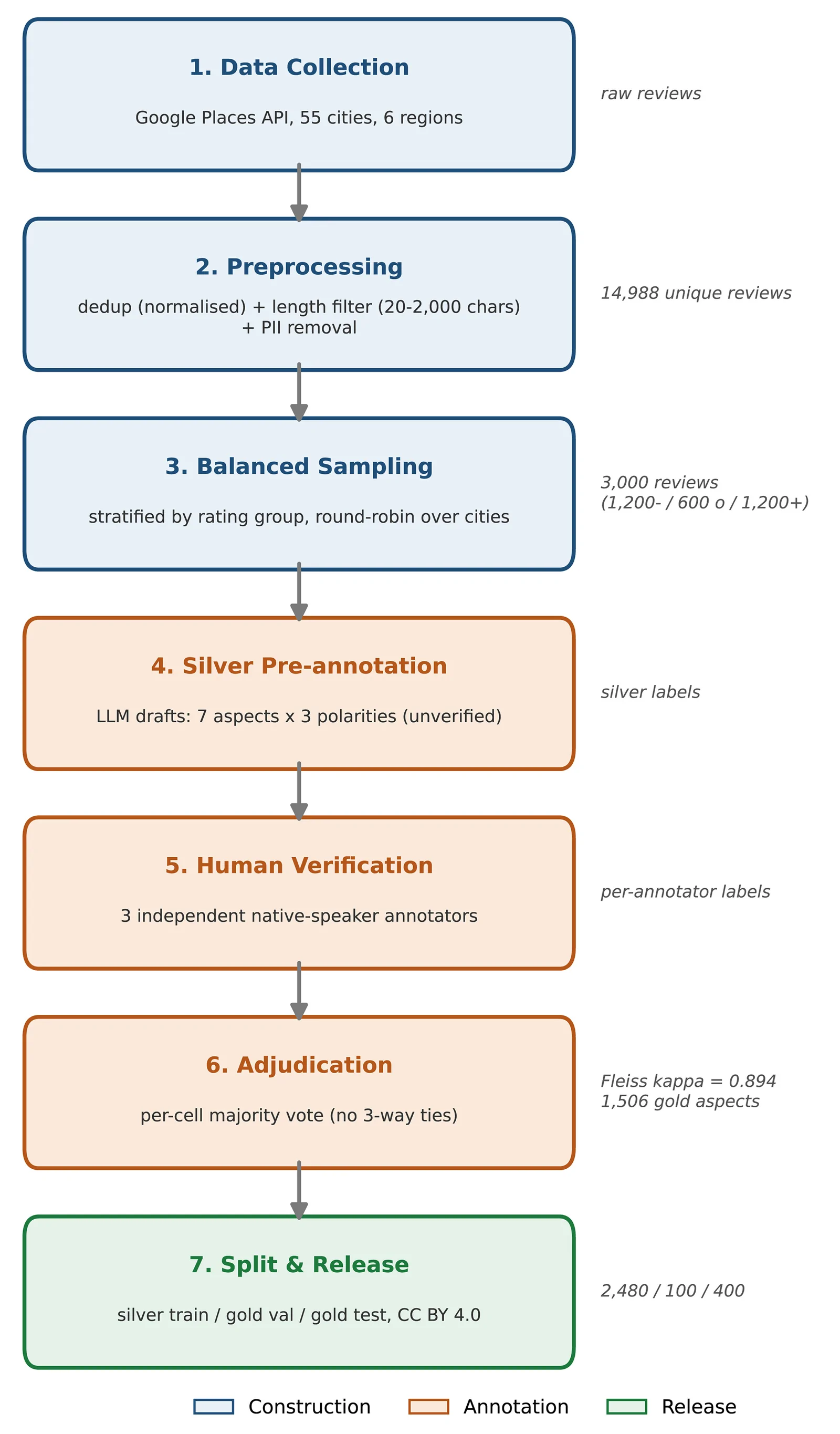
Architecture for the development of the IndoHotelABSA dataset: from Google Places collection and preprocessing, through class-balanced sampling and the two-tier LLM-assisted, human-verified annotation, to the adjudicated silver/gold splits and public release.

Figure 2 summarises the architecture used to develop the dataset, spanning three phases, data construction (collection, preprocessing, balanced sampling), annotation (LLM silver pre-annotation, human verification of a gold subset, and majority-vote adjudication), and release (fixed silver/gold splits under the licence described in the Ethics Statement). Each stage is detailed below. All scripts named in this section are deposited in the code repository. The pipeline was run under CPython 3.14.6 and is standard-library only except for three packages, pinned in requirements.txt: requests 2.32.5 for the Places API calls, google-genai 2.11.0 for the silver pre-annotation, and matplotlib 3.11.0 for Figure 1. Every step after collection and pre-annotation, including sampling, adjudication, agreement, PII scrubbing, and release verification, uses no third-party package at all.

### 4.1 Data collection

Indonesian-language hotel reviews were retrieved on 10 July 2026, in a single session between 02:00 and 05:20 local time (WIB), through the official Google Places API web service: Text Search (/maps/api/place/textsearch/json) for establishment discovery and Place Details (/maps/api/place/details/json, fields=name,review) for review retrieval. Both endpoints were called with language=id, and Place Details additionally with reviews_no_translations=true, so that only original-language review text was returned. Language was therefore constrained at request time rather than by a post-hoc language-identification model.

Because the API caps results per query, each of the 55 cities was queried with eight complementary search expressions, *hotel di {city}, hotel murah di {city}, hotel bintang 5 di {city}, hotel bintang 3 di {city}, penginapan di {city}, guest house di {city}, resort di {city}*, and *hotel dekat pusat kota {city}*, paginated up to three pages per query where a next-page token was returned. The returned establishments were merged across the eight queries and de-duplicated by place identifier, capped at 60 establishments per city, yielding 3,075 distinct hotel entries corresponding to 3,068 distinct establishment names. Place Details returns at most five reviews per establishment, which is the binding constraint on corpus size. Collection produced 15,305 raw reviews.

Filtering and deduplication were deterministic and threshold-free. No fuzzy or embedding-based near-duplicate matching was used. Each text was normalised by collapsing whitespace runs to a single space and stripping leading and trailing whitespace. Reviews shorter than 20 or longer than 2,000 characters were then discarded (169 removed). A deduplication key was formed by lower-casing the normalised text and truncating it to its first 200 characters, and the first review for each distinct key was retained across the pooled corpus (148 removed). This yields the 14,988 unique reviews of the release. The prefix-truncated key implies that two reviews sharing an identical first 200 characters are treated as duplicates even if they diverge later.

Anonymisation was applied at two levels. At collection time, reviewer-identifying *metadata fields*, that is reviewer name, profile and photo URLs, author attribution, and review timestamp, were never requested from the API and were never stored. Place identifiers were used transiently for establishment de-duplication and were likewise not stored. Only review text, star rating, hotel name, and city were written to disk. Because that step does not touch personal data typed by users into the review body, a second, deterministic pass (scrub_pii.py, deposited) was subsequently run over the review text of every released record, replacing telephone numbers, e-mail addresses, and URLs with the typed placeholders [PHONE], [EMAIL], and [URL]. The pass folds text by Unicode NFKC and includes a contextual rule for the homoglyph-obfuscated contact numbers used in listing-owner solicitations, which ordinary digit patterns do not match. It affected 111 of the 14,988 corpus records (0.74%): 109 telephone numbers and 2 URLs, and no e-mail addresses were found. Within the labelled release the pass affected 20 of 2,480 silver records, 1 of 100 validation records, and 3 of 400 test records. Person names appearing in the review body were deliberately not removed. The rationale and the residual risk are discussed in Section 5 and in the datasheet.

### 4.2 Sampling for annotation

Because the raw rating distribution is strongly positively skewed, a random sample would under-represent negative and neutral aspect expressions. We therefore drew a class-balanced sample of 3,000 reviews (sample_for_annotation.py, with the seed fixed at random.seed(42)). Reviews were bucketed by star rating into negative (1–2 stars), neutral (3 stars), and positive (4–5 stars), with target quotas of 40%, 20%, and 40% of n=3,000, that is 1,200, 600, and 1,200 reviews. Within each bucket, reviews were grouped by city, each city list and the city order were shuffled, and reviews were drawn round-robin across the 55 cities until the quota was met. The union was then shuffled and renumbered review_id 1–3,000.

The 500-review gold subset is a random subsample of these 3,000, stratified by rating group so as to preserve the composition of the annotation sample: 206 reviews at 1–2 stars, 105 at 3 stars, and 189 at 4–5 stars. The realised identifiers are released as gold_subset_ids.txt, so that the subset is exactly identifiable and every split is verifiable from the released files.

### 4.3 Aspect schema

Guided by hotel CRM practice, seven non-overlapping aspect categories were defined to jointly cover the guest experience (Section 3). Each mentioned aspect is assigned one of three polarities (positive, negative, neutral). The full scope of each category and the decision rules are provided in annotation_guidelines.md.

### 4.4 Annotation protocol

A two-tier protocol was used: an LLM produced draft labels for all 3,000 sampled reviews (the *silver* tier), and three human annotators verified and corrected a 500-review subset, whose adjudicated labels form the *gold* tier. Human verification was applied to those 500 reviews only. The 2,480 released silver records are unverified model output.

*Silver pre-annotation* Draft labels were produced with Google Gemini (Flash family), accessed through the google-genai Python SDK on 16 July 2026 (preannotate_gemini.py). Because free-tier quotas cap the number of calls per minute and per day, the script is resumable, and the 3,000 drafts were accumulated over several successive runs on that date. Two checkpoints were used across those runs: gemini-2.5-flash, the script default and the checkpoint of most invocations, and gemini-2.0-flash, used for two runs at a modified call spacing. A ten-review trial run at the start used gemini-flash-latest. The script also defines an automatic fallback chain (gemini-2.0-flash-lite, gemini-flash-lite-latest, gemini-2.0-flash, gemini-flash-latest, gemini-2.5-flash-lite) for the case where the requested checkpoint returns a quota error. We report this openly because the serving checkpoint was not recorded per record, so the drafts cannot be attributed individually to one checkpoint. Users reproducing the silver tier should expect checkpoint-level variation and are advised to log the model identifier with each response. No decoding parameters were overridden: all calls used the API defaults for temperature, top-p, top-k, and maximum output tokens. Prompting was zero-shot, with no demonstrations or few-shot examples, one generateContent call per review, 3,000 calls in total, spaced by 4.5 seconds, with up to six retries per review on transport or quota errors (60 s wait on HTTP 429, 15 s on HTTP 503, linear back-off otherwise). The prompt is written in Indonesian, fixes the seven aspects and three polarities as closed lists, requires JSON-only output, and states four decision rules. It is reproduced verbatim in Appendix Appendix A.

Model responses were post-processed deterministically: the first JSON array in the response was extracted by regular expression and parsed. An object was retained only if its aspect was one of the seven schema values *and* its polarity one of positif/negatif/netral. Repeated aspects within one response were deduplicated, keeping the first occurrence, and all other content was discarded. A review whose call failed after six retries was not written and was retried on the next, resumable run, so that all 3,000 reviews were eventually completed. A well-formed but empty array was retained as an empty label set. Twenty-three reviews received such an empty set. Three of them fall in the gold subset and were labelled by the annotators in the normal way, while the remaining 20 lie in the silver pool and were excluded from train_silver.jsonl, since a record with no aspect label carries no supervision signal. Inspection confirms that these 20 mention none of the seven schema aspects: they are generic one-line reactions, bare establishment names, booking-contact solicitations, off-topic commentary, and short non-Indonesian texts.

*Human verification and adjudication* Three annotators, all native Indonesian speakers familiar with the hospitality domain and independent of any model development, each labelled the same 500-review subset. They worked in a purpose-built, self-contained offline HTML application, one copy per annotator, with no network access, no communication between annotators, progress stored locally, and results exported as JSON Lines. Each annotator was briefed with the written guidelines (annotation_guidelines.md) and a worked practice set before starting.

Annotators were *not* blinded to the model output: each aspect row was pre-set to the LLM draft label, and the task was to confirm or correct it, the interface stating this explicitly and instructing annotators not to accept a draft without checking. The protocol is therefore verification of a visible draft rather than independent labelling from scratch, and the agreement statistics reported in Section 3 should be interpreted accordingly (Section 5). Inter-annotator agreement was computed with Fleiss’ κ [25] over all (review, aspect) cells, and pairwise agreement with Cohen’s κ [26]. Both coefficients are defined over the four per-cell categories C={N/A,POSITIF,NEGATIF,NETRAL} and take the form (observed−chance)/(1−chance). For two annotators A and B, Cohen’s κ is

(1) $$\kappa_{\mathrm{Cohen}}=\frac{p_{o}-p_{e}}{1-p_{e}},\;p_{e}=\sum_{c\in C}p_{c}^{A}\;p_{c}^{B},$$

where $p_{o}$ is the proportion of cells that A and B label identically, and $p_{c}^{A}$ and $p_{c}^{B}$ are the marginal proportions with which A and B use category c. For m annotators (here m=3), Fleiss’ κ generalises this to multiple raters,

(2) $$\kappa_{\mathrm{Fleiss}}=\frac{\bar{P}-{\bar{P}}_{e}}{1-{\bar{P}}_{e}},$$

where, over N cells with $n_{ij}$ the number of annotators assigning category j to cell i and $p_{j}=\frac{1}{Nm}\sum_{i=1}^{N}n_{ij}$ the overall proportion of assignments to category j,

$$P_{i}=\frac{1}{m(m-1)}\;\left(\sum_{j\in C}n_{ij}^{2}-m\right),\;\bar{P}=\frac{1}{N}\sum_{i=1}^{N}P_{i},\;{\bar{P}}_{e}=\sum_{j\in C}p_{j}^{2}.$$

Here $P_{i}$ measures the agreement among the m annotators on cell i, $\bar{P}$ is its mean over all cells, and ${\bar{P}}_{e}$ is the agreement expected by chance. Both statistics were computed with compute_kappa.py (deposited). Disagreements were resolved by per-cell majority vote. The adjudication script provides for a tie-break by the LLM label in the event of a three-way tie, but no three-way tie occurred in the 3,500 cells, so that provision was never exercised.

*Blinded re-annotation* Because the non-blinded design bears directly on how the agreement figures should be read, we measured its effect rather than only noting it. A random sub-sample of 100 of the 500 gold reviews (identifiers released as blind_subset_ids.txt) was re-annotated by the same three annotators on 18 August 2026, with every label cleared, no model output shown, and no reference to the earlier round. The interface was rebuilt so that all seven aspects start at n/a.

Over these 700 cells, blinded agreement is Fleiss’ κ=0.975 (97.7% of cells unanimous), against κ=0.908 (91.9%) for the same reviews under the original protocol. Agreement is therefore *higher* without the draft, so the reported κ is not an artefact of three annotators accepting one shared suggestion. Since agreement that high would otherwise be more consistent with annotators working together than with a sound protocol, the returned files were checked for independence: the three chose the same aspect set on 94 of the 100 reviews but recorded them in the same order on only 66 of those, and label order follows the on-screen aspect order in 88, 89, and 86 reviews respectively, as expected when each annotator works down an empty form, against 30, 28, and 31 in the draft-anchored round, where order instead reflects which cells were edited.

The blinded labels nevertheless differ from the adjudicated gold in 133 of 697 cells (19.1%), and the divergence is directional. The blinded round marks 378 aspect mentions against 303 in the gold (+24.8%), and 65% of the differing cells are aspects the gold left unmarked, 60 of those 87 being negative. The visible draft therefore appears to have depressed aspect *recall* rather than inflated agreement: annotators working from an empty form detect mentions that the draft omitted and that the verification pass did not restore. Users should treat the gold aspect inventory as conservative, particularly for negative mentions, and the polarity labels attached to detected aspects as the more reliable part of the resource. Because the same annotators had seen these reviews three weeks earlier, recall of the first session cannot be excluded. It would, however, pull the blinded labels towards the gold, whereas the observed divergence is away from it. The three blinded label files, the sub-sample identifiers, and the analysis script are released so that this comparison can be reproduced and extended.

To make these statistics independently reproducible, the release includes each annotator’s raw, unadjudicated label file, the per-cell adjudication record, the LLM draft that annotators saw, and the identifiers of the gold subset (Table 1), together with the scripts compute_kappa.py and adjudicate_gold.py that regenerate Table 3 and gold_final.jsonl from them. The released corpus distinguishes silver from gold labels record by record, and the dataset is documented with a datasheet following the *Datasheets for Datasets* framework [24].

## Limitations

Reviews originate from a single mapping platform’s public API. Although coverage spans 3,075 establishments in 55 cities, platform-specific reviewer demographics and moderation may introduce selection bias. The annotated sample is deliberately rating-balanced (40/20/40) rather than prevalence-representative, so aspect and polarity frequencies in the annotated tiers must not be read as population estimates for Indonesian hotel reviews. Users needing prevalence-representative estimates should re-sample from dataset_merged.jsonl, whose rating distribution is reported in Section 3. Only a 3,000-review sample is annotated, and the seven CRM aspects are fixed a priori, so implicit or emerging aspects outside the schema are not captured. Silver labels are LLM-drafted and should not be treated as ground truth, and only the 500 gold reviews are human-verified.

The LLM draft was visible during verification, and the blinded re-annotation reported in Section 4.4 shows what this cost. Agreement is not the casualty: blinded κ on the re-annotated sub-sample is higher, not lower. The cost falls on aspect detection. Working from an empty form, the same annotators mark about a quarter more aspect mentions than the gold contains, and two-thirds of the divergent cells are aspects the gold leaves unmarked, mostly negative ones. The gold aspect inventory should therefore be treated as conservative: a model that predicts an aspect the gold does not record may be right rather than wrong, and recall measured against this gold is likely understated, particularly for negative mentions. Polarity assignment on aspects that are detected is the more dependable part of the resource. This limitation is inherited by the silver tier, which was produced by the same draft process without any human pass at all, and quantifying it across the full 500-review gold set, or re-annotating the corpus blind, remains open work.

The neutral class is rare in both tiers, at 4.6% of gold aspect annotations (69 of 1,506) and 4.6% of silver ones (334 of 7,338). The agreement of the two rates, together with the low correction rate on the gold subset, indicates that the scarcity is a property of the review genre and of our narrow definition of netral as factual mention without evaluation, rather than a bias introduced by the LLM pre-annotation: platform reviews are volunteered and star-anchored, so a guest who mentions an aspect at all usually does so in order to praise or complain, and purely descriptive aspect mentions are uncommon. The skew of the underlying rating distribution (Figure 1) contributes but is not sufficient as an explanation, since sampling deliberately over-weighted 3-star reviews to 20% of the annotation sample and neutral annotations remained rare even so. Practically, per-class neutral metrics rest on few instances and should be reported with confidence intervals. Users targeting the neutral class may wish to over-sample 3-star reviews from the unlabelled corpus or to treat neutral detection as a distinct low-resource sub-task. Whether a broader neutral definition, admitting mixed or hedged evaluation, would recover more instances is left to future work.

Two residual data-quality caveats should be noted. First, because language was constrained at request time rather than verified afterwards, a small minority of English or regional-language reviews written by users remains in the corpus. Second, the in-text anonymisation pass (Section 4.1) targets telephone numbers, e-mail addresses, and URLs but deliberately leaves person names in place: Indonesian named-entity recognition over informal, code-mixed review text has a high false-positive rate on hotel, place, and brand names, and destructive substitution would damage precisely the aspect-bearing spans the dataset exists to support. Person names occurring in this corpus are overwhelmingly staff first names used in praise, which are analytically relevant and low-risk. The takedown procedure described in the Ethics Statement covers any request for removal.

Establishment names are likewise retained rather than pseudonymised. We considered replacing them with opaque identifiers, but rejected this for three reasons: the establishment name is a factual datum and is not the material whose redistribution status is at issue, per-property aggregation is one of the CRM uses the dataset is intended to support, and pseudonymisation could not be applied consistently, because 21.1% of reviews name their own establishment inside the review text (4.6% give the full name verbatim), so masking the metadata field alone would suggest a protection the release does not provide. Users should note that the corpus therefore contains strongly negative, occasionally accusatory opinions attached to identifiable businesses. These are the subjective statements of individual reviewers, are reproduced without endorsement, and must not be used to disparage or harass any property. The same takedown procedure applies.

## Ethics Statement

The reviews were collected through the official Google Places API. No scraping was used. Collection and redistribution are treated as separate questions. The review texts are secondary, user-generated content: copyright in each review remains with its author, and the content is served subject to the platform’s terms, so the authors neither hold nor claim the right to relicense it. The release therefore carries a split licence. The authors’ own contribution, that is the aspect–polarity annotations, the aspect schema and annotation guidelines, the split definitions, the datasheet, the summary statistics, and all code, is released under CC BY 4.0. The review text and hotel names are redistributed as third-party content for non-commercial academic research only, with attribution to the source platform and to the individual review authors, and are not relicensed. LICENSE_DATA.md states this, reproduces the applicable attribution requirement, and documents a takedown procedure: a named contact, and removal of any record within 30 days on request from the platform or from a review author, followed by a versioned re-release.

Only permitted, non-personal fields were retained. Reviewer names, profile and photo URLs, author attributions, and timestamps were never requested from the API and were never stored, and place identifiers were used transiently for de-duplication only. In addition, telephone numbers, e-mail addresses, and URLs were removed from the review text itself (Section 4.1). No attempt is made to re-identify reviewers, and no private data were accessed. The deposit is a static research corpus of short texts. It provides no lookup, mapping, or place-discovery functionality and is not a substitute for the source API. Users remain subject to the source platform’s terms.

A large language model was used to produce draft (silver) annotations for all 3,000 sampled reviews. Human verification was applied to the 500-review gold subset only: those 500 reviews were checked and corrected independently by three annotators and adjudicated by majority vote. The remaining 2,480 released silver records are unverified model output and are labelled as such in every released file. This work did not involve experiments on humans or animals.

## Ethics Declaration

The author(s) declare(s) that the study does not involve humans nor animal subjects.

## CRediT authorship contribution statement

**Xaverius Sika:** Conceptualization, Methodology, Software, Investigation, Data curation, Writing – original draft. **Eko Sediyono:** Conceptualization, Supervision, Writing – review & editing. **Irwan Sembiring:** Supervision, Validation, Writing – review & editing. **Teguh Wahyono:** Supervision, Validation, Writing – review & editing. **Eko Arip Winanto:** Software, Validation, Writing – review & editing.

## Appendix A Silver Pre-Annotation Prompt

The following prompt was sent verbatim, once per review, with {text} replaced by the review text. It is also distributed in annotation_guidelines.md. Anda anotator Aspect-Based Sentiment Analysis untuk ulasan hotel berbahasa Indonesia. Untuk ulasan di bawah, keluarkan HANYA JSON: daftar objek {”aspect”: <aspek>, ”polarity”: <sentimen>}. Aspek valid (persis): [’Lokasi’, ’Kebersihan’, ’Pelayanan’, ’Kamar & Fasilitas’, ’Harga’, ’Makanan & Minuman’, ’Fasilitas Pendukung’]. Sentimen valid: [’POSITIF’, ’NEGATIF’, ’NETRAL’]. Aturan: - Hanya sertakan aspek yang benar-benar disebut. - Perhatikan negasi (mis. ’tidak bersih’ = NEGATIF). - ’Kamar & Fasilitas’ = fasilitas DALAM kamar; ’Fasilitas Pendukung’ = fasilitas UMUM (wifi, parkir, kolam). - Bila faktual tanpa penilaian, polaritas NETRAL. - Jangan beri penjelasan apa pun di luar JSON. Ulasan: ”{text}” Jawaban (JSON): In English: *“You are an Aspect-Based Sentiment Analysis annotator for Indonesian-language hotel reviews. For the review below, output ONLY JSON: a list of objects {“aspect”: <aspect>, “polarity”: <sentiment>}. Valid aspects (exactly): [...]. Valid sentiments: [...]. Rules: include only aspects that are actually mentioned; watch for negation (e.g. ‘tidak bersih’ = NEGATIF); ‘Kamar & Fasilitas’ means facilities INSIDE the room, ‘Fasilitas Pendukung’ means SHARED facilities (wifi, parking, pool); if a mention is factual without evaluation, the polarity is NETRAL; do not give any explanation outside the JSON.”*

## Appendix B Supplementary Materials

Supplementary Data S1 Supplementary Raw Research Data. This is open data under the CC BY license http://creativecommons.org/licenses/by/4.0/ Supplementary Data S1
