# Supplementary material for: IndoHotelABSA: A large-scale aspect-based sentiment analysis dataset of Indonesian hotel reviews for hospitality CRM
Source: Data Brief. 2026 Sep 3;69:113209. doi: 10.1016/j.dib.2026.113209 (PMC13599638; doi:10.1016/j.dib.2026.113209)
Supplement: Supplementary file 1 [file mmc1.pdf]

IndoHotelABSA: An Explainable Aspect-Based Sentiment Analysis Framework and Benchmark Dataset for Indonesian Hotel Reviews in Hospitality CRM

Xaverius Sika[a,b,*], Eko Sediyono[b], Irwan Sembiring[b], Teguh Wahyono[b] and Eko Arip Winanto[c]

[a]Department of Information System, Universitas Dinamika Bangsa, Jambi, Indonesia
[b]Faculty of Information Technology, Satya Wacana Christian University (UKSW), Salatiga, Indonesia
[c]Department of Computer Engineering, Universitas Dinamika Bangsa, Jambi, Indonesia

ARTICLE INFO

Keywords:
Aspect-based sentiment analysis
Indonesian NLP
Low-resource
Large language models
Hotel reviews
Explainable CRM
Benchmark dataset

ABSTRACT

Online reviews are the richest voice-of-customer source for hospitality customer relationship management (CRM), yet aspect-based sentiment analysis (ABSA) of Indonesian hotel reviews is held back by the lack of a large-scale, quality-controlled benchmark. We make three contributions. First, we release **IndoHotelABSA**, the first large-scale Indonesian hotel ABSA benchmark: 14,988 reviews across 55 cities and all six major regions, with a 500-review gold subset annotated by three independent annotators for seven CRM-meaningful aspect categories at almost-perfect agreement (Fleiss' $\kappa = 0.894$). Second, we benchmark classical, encoder, generative, and large language model (LLM) methods, with three findings: Indonesian-specific pre-training is decisive (IndoBERT exceeds multilingual encoders by over eight Joint-F1 points); LLM effectiveness is scale-dependent, as a zero-shot large LLM wins while a LoRA-tuned small LLM loses to encoders, so the advantage stems from scale and the zero-shot regime rather than the LLM paradigm; and the neutral class is the principal unsolved sub-problem, on which all fine-tuned models collapse. A cross-benchmark check on HoASA reproduces the published state of the art, validating our pipeline. Third, we present an explainable CRM framework with an aspect-aware model, HI-ABSA. Rather than a state-of-the-art accuracy claim, HI-ABSA is an operationally credible, CRM-preferred alternative: statistically on par with the strongest local encoder while uniquely adding native per-aspect interpretability, on-premise privacy, and zero per-inference cost. Its aspect scores drive an actionable dashboard that, with our prior cancellation-risk model, forms a dual-signal (Integrated Customer Data Analysis) view of hospitality CRM.

1. Introduction

Online guest reviews have become the primary channel through which hotels receive continuous, unsolicited feedback about their service. For hospitality customer relationship management (CRM), these reviews constitute the richest available source of voice-of-customer information: they reveal not only whether a guest was satisfied, but *why*. Prior work has established that review-derived signals are strongly associated with tourist satisfaction and revenue outcomes (Guo et al., 2017). Because such feedback now arrives continuously and at a volume no manager can read by hand, hotels increasingly depend on automated analysis to turn it into timely, aspect-level operational decisions, this is a core CRM capability rather than an optional analytic. Translating this unstructured text into operational insight, however, remains difficult, because a single review typically mixes praise and complaint across several distinct facets of the guest experience.

Document-level sentiment analysis, which assigns one polarity label to an entire review, is ill-suited to this task. A review such as "the location is perfect but the room was dirty and the staff were slow" carries a positive, a negative, and a second negative signal that a single document-level label necessarily collapses. Aspect-based sentiment analysis (ABSA) was introduced precisely to recover this structure, decomposing a text into (aspect, polarity) pairs (Pontiki et al., 2014, 2016; Zhang et al., 2023). For hospitality CRM, the aspect dimension is not incidental detail but the actionable unit: a revenue manager needs to know *which* operational domain, cleanliness, service, pricing, is driving dissatisfaction, in order to intervene.

Research on ABSA has matured rapidly. The SemEval shared tasks fixed its canonical formulation and evaluation benchmarks (Pontiki et al., 2014, 2016), after which fine-tuned BERT-style encoders (Sun et al., 2019) and, more recently, generative and instruction-tuned formulations (Zhang et al., 2021; Scaria et al., 2024) became dominant, while a parallel line probes large language models as zero- and few-shot predictors (Wang et al., 2023; Zhang et al., 2024). This progress is, however, overwhelmingly English-centric. Fine-grained ABSA has only recently reached other low-resource languages, for example Bengali aspect sentiment triplet extraction (Islam et al., 2026), Arabic dataset creation with explainability (Al Saidat et al., 2026), and large language model studies for Swahili and code-switched Arabic (Mgeni, 2026; Sabty et al., 2026). For Indonesian, IndoNLU introduced the IndoBERT family and the HoASA hotel dataset (Wilie et al., 2020), but HoASA is drawn from a single platform, is limited in scale, and uses a platform-specific

*Corresponding author.
Email addresses: xaver_ius@unama.ac.id (X. Sika); eko@uksw.edu (E. Sediyono); irwan@uksw.edu (I. Sembiring); teguh.wahyono@uksw.edu (T. Wahyono); winanto@unama.ac.id (E.A. Winanto)
ORCID(s): 0009-0005-0279-7863 (X. Sika); 0000-0001-6034-9659 (E. Sediyono); 0000-0002-6625-7533 (I. Sembiring); 0000-0002-5433-5952 (T. Wahyono); 0000-0003-3274-1885 (E.A. Winanto)

aspect inventory. In hospitality specifically, review mining has largely stopped at descriptive or topic-level analysis of satisfaction and revenue (Guo et al., 2017), without aspect-level polarity mapped to concrete CRM actions. Across method, language, and application, therefore, prior work leaves Indonesian hotel ABSA for CRM under-served.

Indonesian, one of the most widely spoken languages in the world and the dominant language of a rapidly growing Southeast Asian tourism market, is comparatively low-resource: there is, to our knowledge, no publicly available, human-annotated ABSA benchmark for Indonesian hotel reviews. This absence has two consequences. First, it prevents rigorous, reproducible comparison of methods on Indonesian hospitality text. Second, it forces practitioners to rely on English-trained models or coarse document-level tools that do not transfer cleanly to Indonesian review language, which is informal, code-mixed, and rich in domain-specific expressions.

At the same time, the methodological landscape is shifting rapidly. Pre-trained language models such as multilingual BERT and XLM-RoBERTa (Devlin et al., 2019; Conneau et al., 2020), and Indonesian-specific models such as those released with IndoNLU (Wilie et al., 2020), have raised the bar for low-resource understanding, while large language models (LLMs) promise strong few-shot performance without task-specific training (Brown et al., 2020; Touvron et al., 2023). Yet it is not established which of these paradigms, fine-tuned encoders, fine-tuned LLMs, or few-shot prompted LLMs, is most effective and cost-efficient for Indonesian hotel ABSA, nor how their predictions translate into decision-support value for CRM.

We identify three specific gaps. **(G1)** Existing Indonesian hotel ABSA data (notably HoASA in IndoNLU (Wilie et al., 2020)) is drawn from a single platform, limited in scale, and uses a platform-specific aspect inventory; no large-scale, nationwide, CRM-aligned benchmark exists. **(G2)** No systematic benchmark compares classical, encoder-based, and LLM-based ABSA methods for this low-resource setting, and no aspect-aware architecture has been proposed for it. **(G3)** ABSA outputs are rarely connected to an explicit, actionable CRM decision framework in the hospitality domain.

To address these gaps, this paper makes the following contributions, led by the benchmark resource:

1. **IndoHotelABSA, a large-scale benchmark dataset (primary contribution).** We construct and publicly release a corpus of 14,988 Indonesian hotel reviews spanning 55 cities across all six major regions, with a 500-review gold subset independently annotated by three annotators for seven CRM-meaningful aspect categories and three-way polarity at almost-perfect agreement (Fleiss' $\kappa = 0.894$), under a documented, transparently-disclosed LLM-assisted, human-verified protocol. It is an order of magnitude larger and geographically broader than the prior HoASA resource.
2. **A comprehensive empirical benchmark with new findings.** We evaluate classical, encoder-based, generative, and LLM-based methods and establish three findings for Indonesian hotel ABSA: (i) Indonesian-specific pre-training is decisive; (ii) LLM effectiveness is scale-dependent, a large zero-shot LLM excels while a small LoRA-fine-tuned LLM underperforms encoders, so the advantage stems from scale and the zero-shot regime rather than the LLM paradigm itself; and (iii) the neutral class is the principal unsolved sub-problem, on which every fine-tuned model collapses. Findings are supported by paired-bootstrap and McNemar significance testing, an explicit anchoring analysis, and a cross-benchmark validation on HoASA that reproduces the published state of the art, confirming our pipeline is correctly calibrated.
3. **An explainable CRM framework with a deployment-oriented model.** We present a dashboard that converts aspect-level sentiment into actionable, interpretable managerial priorities and, connecting to our prior booking-cancellation model (Sika et al., 2026), a dual-signal *Integrated Customer Data Analysis* (ICDA) view of hospitality CRM in which this paper contributes the perceptual (sentiment) pillar. As part of it we propose HI-ABSA. We deliberately position HI-ABSA not as a state-of-the-art accuracy claim but as an *operationally credible, CRM-preferred alternative*: it is statistically on par with the strongest locally-deployable encoder while uniquely providing native per-aspect interpretability, on-premise privacy, and zero per-inference cost, precisely the properties a real CRM deployment requires but that the more accurate third-party LLM lacks.

These contributions are organised around four research questions. **RQ1:** How reliably can large-scale Indonesian hotel ABSA be annotated, and what inter-annotator agreement is achievable? **RQ2:** Which method paradigm (classical, encoder, or LLM) is most effective for this low-resource task, and how does model scale mediate the outcome? **RQ3:** Does aspect-aware modelling add value, in accuracy or interpretability? **RQ4:** How can aspect-level sentiment be operationalised into an actionable CRM decision framework?

The remainder of this paper is organised as follows. Section 2 reviews related work. Section 3 describes the IndoHotelABSA dataset and its annotation. Section 4 details the benchmarked methods. Section 5 reports experimental results. Section 6 discusses the findings. Section 7 presents the explainable CRM framework. Section 8 notes limitations, and Section 9 concludes.

2. Related Work

2.1. Aspect-Based Sentiment Analysis

Sentiment analysis has matured from lexicon and classical machine-learning methods into deep-learning-based approaches (Medhat et al., 2014; Zhang et al., 2018; Birjali

et al., 2021). Aspect-based sentiment analysis decomposes opinionated text into finer-grained units than document-level sentiment (Liu, 2012). The SemEval-2014, 2015, and 2016 shared tasks established the canonical formulation and evaluation benchmarks, covering aspect-term extraction, aspect-category detection, and aspect-category polarity across the restaurant and laptop domains (Pontiki et al., 2014, 2015, 2016). Neural approaches then became dominant: fine-tuning BERT, via auxiliary-sentence construction (Sun et al., 2019), end-to-end tagging (Li et al., 2019), or direct classification (Hoang et al., 2019), yields strong ABSA performance. A subsequent line of work recasts ABSA as sequence generation, unifying its sub-tasks in a single text-to-text model (Zhang et al., 2021; Yan et al., 2021), culminating in instruction-tuned formulations such as InstructABSA (Scaria et al., 2024). The survey by Zhang et al. (2023) organise this task space and its methods, while Nazir et al. (2022) catalogue the open challenges. Most of this work, however, targets English benchmarks, leaving low-resource languages, and domain-specific hospitality text in particular, comparatively underexplored. Recent efforts have begun to extend fine-grained ABSA to other low-resource languages, such as Bengali aspect sentiment triplet extraction (Islam et al., 2026), yet Indonesian hospitality reviews remain without a large-scale, quality-controlled benchmark.

2.2. Transformer and Large Language Models for Sentiment

The Transformer architecture (Vaswani et al., 2017) and the pre-train/ fine-tune paradigm of BERT (Devlin et al., 2019) became the dominant approach to sentiment classification, including ABSA. For multilingual and low-resource settings, cross-lingual encoders such as XLM-RoBERTa (Conneau et al., 2020) provide transferable representations without language-specific pre-training from scratch. More recently, instruction-tuned large language models (Brown et al., 2020; Ouyang et al., 2022; Touvron et al., 2023) have demonstrated strong few-shot and zero-shot capabilities, raising the question of whether task-specific fine-tuning is still necessary for structured tasks such as ABSA. Early probes such as Wang et al. (2023) found LLMs competitive on coarse sentiment but weaker on fine-grained aspects, and a broader reality check by Zhang et al. (2024) finds that, while LLMs excel at simpler sentiment tasks in zero/few-shot settings, they still lag fine-tuned models on fine-grained, structured tasks such as ABSA, especially where labelled data exists. In low-resource and code-switched settings specifically, recent studies probe LLMs directly, including prompt-based few-shot learning for Arabic and code-switched text (Sabty et al., 2026), the use of large language models for Swahili (Mgeni, 2026), and explainable generative sentiment analysis (Dharshini and Mahendran, 2026). This trade-off between fine-tuned encoders and prompted LLMs, in accuracy, cost, and robustness, remains actively debated and, for low-resource Indonesian hospitality text, largely untested.

2.3. Indonesian and Low-Resource NLP

Indonesian NLP has advanced substantially with the release of large-scale benchmarks and pre-trained models. IndoNLU (Wilie et al., 2020), IndoLEM (Koto et al., 2020), and IndoNLG (Cahyawijaya et al., 2021) introduced downstream-task suites and the IndoBERT family of models, later extended to social-media text by IndoBERTweet (Koto et al., 2021). Broader efforts such as NusaCrowd (Cahyawijaya et al., 2023) and NusaX (Winata et al., 2023) consolidated Indonesian and regional-language resources, including sentiment data. For aspect-based sentiment specifically, the IndoNLU benchmark (Wilie et al., 2020) includes two aspect-level datasets: CASA (automobile reviews) and **HoASA**, a hotel aspect-based sentiment dataset built from a single online travel platform. HoASA is the closest prior resource to our work and an important reference point; however, it is drawn from one platform, is limited in scale, and uses a platform-specific aspect inventory not aligned with CRM decision dimensions. Table 1 positions IndoHotelABSA against these resources: relative to HoASA, our corpus is an order of magnitude larger, spans 55 cities across all six major Indonesian regions from a national mapping source, adopts a CRM-oriented seven-aspect schema, and is released together with an explainable-CRM framework and a new aspect-aware model. Informal review language, replete with colloquialisms, code-switching, and non-standard spelling, further motivates a domain-specific, larger-scale resource. Indonesian NLP is at the same time expanding into applied domains, such as named-entity recognition for disaster information management (Shidik et al., 2026). More broadly, across low-resource languages a recurring and productive pattern pairs the creation of a new, quality-controlled dataset with model explainability (Al Saidat et al., 2026), precisely the pattern this paper follows for Indonesian hotel ABSA.

2.4. Sentiment Analysis in Hospitality and Tourism CRM

Within hospitality and tourism, review mining has been used to model tourist satisfaction and to inform revenue management. Guo et al. (2017), for example, applied topic modelling to online ratings and reviews to extract dimensions of tourist satisfaction. Such studies establish the business value of review-derived signals, but they typically stop at descriptive or topic-level analysis and do not provide aspect-level polarity predictions that map onto operational CRM actions, nor do they connect qualitative review signals with quantitative behavioural outcomes such as booking cancellation. Our framework is designed to close this practical gap and, in conjunction with our prior churn-prediction work (Sika et al., 2026), to support a dual-signal view of hospitality CRM.

2.5. Research Gap

Synthesising the above, three gaps motivate this work: the absence of a public, quality-controlled Indonesian hotel ABSA benchmark (G1); the lack of a systematic comparison of classical, encoder-based, and LLM-based methods for this

Table 1
IndoHotelABSA in the context of Indonesian sentiment datasets. SmSA, CASA, and HoASA are from the IndoNLU benchmark (Wilie et al., 2020). Doc. = document-level; ABSA = aspect-based; prior-dataset sizes are approximate.

Dataset	Domain	Task	#Inst.	Coverage	CRM
SmSA	general	Doc. sentiment	∼12,760	–	no
CASA	automobile	ABSA	∼1,000	1 source	no
HoASA	hotel	ABSA	∼2,300	1 platform	no
**IndoHotelABSA**	hotel	ABSA	**14,988**	**55 cities**	**yes**

Table 2
Qualitative positioning of this study against the most relevant prior work. ABSA = aspect-based sentiment analysis; ASTE = aspect sentiment triplet extraction; enc. = fine-tuned encoder.

Study	Task / language	Model family	Explainability	Main limitation relative to this study
Guo et al. (2017)	Review mining / hotel (multi-ling.)	Topic modelling	None	Descriptive only; no aspect-level polarity; no behavioural link
Wilie et al. (2020) (HoASA)	Hotel ABSA / Indonesian	IndoBERT (enc.)	None	Single platform; ∼2.3k reviews; platform-specific aspects; not CRM-aligned
Islam et al. (2026)	ASTE / Bengali	BiLSTM–CRF	None	Different language; product reviews; no CRM dashboard
Al Saidat et al. (2026)	Smishing / Arabic	CNN–BiGRU	LIME (post hoc)	Different task (binary detection); not aspect-based
**This study**	**Hotel ABSA / Indonesian**	**IndoBERT + HI-ABSA + LLMs**	**Native attention + CRM dashboard**	**14,988-review corpus; CRM schema; benchmark + framework**

low-resource task (G2); and the limited connection between ABSA outputs and actionable, explainable CRM decision support (G3). Table 2 positions this study against the most relevant prior work along the task, model, explainability, and limitation axes, making explicit that no single prior study combines an Indonesian hotel ABSA corpus, a systematic method benchmark, and an explainable CRM framework. The following sections describe the dataset, methods, and framework through which we address these gaps.

3. The IndoHotelABSA Dataset

This section describes the construction of **IndoHotelABSA**, a benchmark corpus of Indonesian hotel reviews annotated for aspect-based sentiment. We detail data collection, the aspect schema, the annotation protocol and its quality control, and summary statistics; the released corpus is accompanied by a datasheet documenting its provenance and intended use (Gebru et al., 2021).

3.1. Data Collection

Reviews were collected from the Google Places API, an official and terms-compliant source, covering 55 cities spanning all major regions of Indonesia (Sumatra, Java, Bali and Nusa Tenggara, Kalimantan, Sulawesi, and Maluku–Papua). To maximise the diversity of hotels retrieved beyond the API's per-query result cap, each city was queried with several complementary search expressions (e.g., budget, mid-range, and upscale hotels, guest houses, and resorts), and the resulting establishments were merged and de-duplicated by place identifier. For every hotel, publicly available Indonesian-language reviews were retrieved. Reviewer names and any personally identifying metadata were discarded at collection time; only the review text, star rating, hotel name, and city were retained. Data were gathered through the official Google Places API in accordance with its terms of use, and the resulting corpus is released for non-commercial research under a CC BY 4.0 licence; users of the corpus remain subject to the source platform's terms. No private data or non-public content was accessed, and no attempt is made to re-identify reviewers.

After removing exact and near-duplicate texts (normalised, case-insensitive) and reviews shorter than 20 or longer than 2,000 characters, the raw collection yielded **14,988 unique reviews** with a mean length of 348 characters (median 250). The corpus spans all six major regions of Indonesia, with Java (4,978 reviews), Sumatra (3,412), and Bali–Nusa Tenggara (2,428) most represented, followed by Kalimantan (1,585), Sulawesi (1,564), and Maluku–Papua (1,021). Figure 1 summarises the star-rating distribution, review-length distribution, and regional coverage; the rating distribution is positively skewed, as is typical of online review platforms, motivating the balanced sampling described next. Table 3 summarises the preprocessing pipeline and the rationale for each step; the guiding principle is to remove noise and duplication without erasing the informal, code-mixed cues that carry aspect-level sentiment.

Table 3
Preprocessing pipeline for IndoHotelABSA and its rationale. The design deliberately favours minimal normalisation to preserve authentic low-resource review signal.

Step	Operation	Rationale
Deduplication	Remove exact and near-duplicate texts (normalised, case-insensitive)	Prevents train/test leakage and popularity bias from repeated reviews
Length filtering	Drop reviews shorter than 20 or longer than 2,000 characters	Removes empty/spam entries and length outliers with no reliable aspect signal
PII removal	Discard reviewer names and identifying metadata at collection time	Privacy by design; only review text, star rating, hotel name, and city are retained
Minimal normalisation	Keep informal spelling, colloquialisms, and code-mixing verbatim	Over-normalisation erases the aspect and sentiment cues typical of Indonesian review language
Tokenization	IndoBERT WordPiece, maximum length 192 tokens	Subword units handle out-of-vocabulary and informal Indonesian words
Class-balanced sampling	Stratify the 3,000-review annotation sample by rating group, round-robin over 55 cities	Guarantees sufficient negative and neutral aspect evidence for training and evaluation

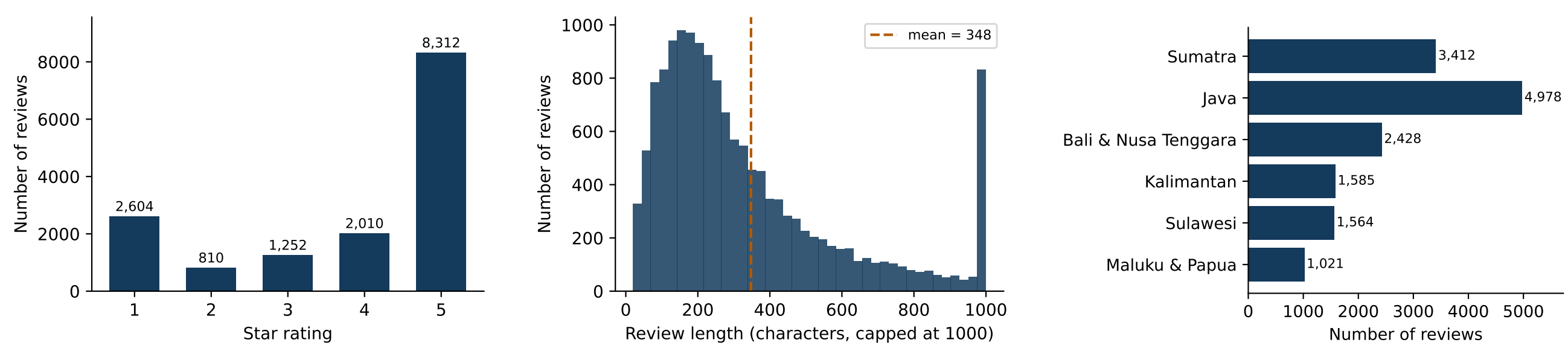

(a) Star-rating distribution (b) Review-length distribution (c) Coverage by region

Figure 1: Descriptive statistics of the 14,988-review IndoHotelABSA corpus: (a) star ratings are positively skewed; (b) most reviews are short-to-medium length (mean 348 characters); (c) coverage spans all six major Indonesian regions.

3.2. Sampling for Annotation

Annotating all 14,988 reviews is impractical, and a random sample would inherit the strong positive skew, under-representing negative aspect expressions that are most valuable for both training and CRM action. We therefore drew a **class-balanced annotation sample of 3,000 reviews**, stratified by rating group into 1,200 negative (1–2 stars), 600 neutral (3 stars), and 1,200 positive (4–5 stars), and spread evenly across all 55 cities via round-robin selection. This balanced design ensures sufficient negative and neutral examples for every aspect category.

3.3. Aspect Categories

Guided by hotel CRM practice, we define seven non-overlapping aspect categories that jointly cover the guest experience (Table 4). Each mentioned aspect is assigned one of three polarities: positive, negative, or neutral.

3.4. Annotation Protocol

To make large-scale annotation tractable while preserving human authority over the gold labels, we adopt a two-tier, *LLM-assisted, human-verified* protocol.

Table 4
The seven aspect categories of IndoHotelABSA.

Aspect	Scope
Location	access, proximity to attractions/transport
Cleanliness	room, bathroom, linen, odour, pests
Service/Staff	friendliness, speed, check-in/out, professionalism
Room & Facilities	in-room size, comfort, AC, TV, water heater
Price/Value	affordability, value for money
Food & Beverage	breakfast, restaurant, taste, variety
Public Facilities	Wi-Fi, parking, pool, gym, lift, lobby

Silver tier (LLM pre-annotation). Each sampled review is first pre-annotated by a large language model prompted, in Indonesian, to output aspect–polarity pairs restricted to the seven-category schema. These machine labels are

Table 5
IndoHotelABSA gold-set annotation quality.

Metric	Value
Number of annotators	3
Gold reviews (human-verified)	500
Multiply-annotated subset	500 (identical for all three)
Overall Fleiss' κ	0.894
Mean per-aspect κ	0.881
Three-way ties in adjudication	0
LLM–human agreement rate	92.1%

Table 6
Per-aspect Fleiss' κ over the 500-review gold subset.

Aspect	Fleiss' κ
Location	0.811
Cleanliness	0.872
Service/Staff	0.906
Room & Facilities	0.892
Price/Value	0.905
Food & Beverage	0.922
Public Facilities	0.859
Overall	0.894

treated strictly as *drafts*; every record is flagged as machine-generated and unverified.

Gold tier (human verification). Three human annotators, all native Indonesian speakers familiar with the hospitality domain and independent of the model-development process, then independently reviewed the *same* 500-review subset, correcting the aspect–polarity assignments where necessary through a purpose-built, offline verification interface distributed to each annotator. Before starting, each annotator was briefed on the seven-category schema and the decision rules of the annotation guidelines. The human decision is final; the LLM draft only accelerates the process and never substitutes for human judgement. Inter-annotator agreement is measured with Fleiss' κ (Cohen, 1960) over all (review, aspect) cells; disagreements are resolved by per-cell majority vote (no three-way ties occurred). This design is disclosed transparently: the released corpus distinguishes LLM-drafted (silver) from human-verified (gold) labels, and all reported test evaluations use the human-verified gold labels.

3.5. Annotation Quality

Table 5 summarises the annotation quality of the gold set. Overall agreement across the three annotators reaches Fleiss' $\kappa = 0.894$ ("almost perfect"), with per-aspect κ ranging from 0.811 (Location) to 0.922 (Food & Beverage), all well above the 0.70 target (Table 6). This figure is measured *before* adjudication, so it reflects genuine independent agreement rather than post-hoc reconciliation; the pairwise Cohen's κ between annotators ranges from 0.846 to 0.987 (raw agreement 90.9–99.3%). In addition to κ, we report the *LLM–human agreement rate*: 92.1% of LLM-drafted (review, aspect) cells were retained unchanged after adjudicated human verification, while 7.9% were corrected. This quantifies both the effectiveness of LLM pre-annotation as an accelerator *and* the residual, non-negligible need for human verification in the LLM-assisted protocol. The adjudicated gold set contains 1,506 aspect annotations (727 negative, 710 positive, 69 neutral), confirming that the balanced sampling of Section 3.2 yields ample negative evidence.

3.6. Representative Examples

Table 7 shows representative gold-annotated reviews, quoted verbatim (including original informal spelling), with English glosses for non-Indonesian readers. The examples illustrate the phenomena that motivate the resource: a single review frequently mixes polarities across several aspects (Example 1 carries positive, negative, and neutral aspects simultaneously), negative sentiment is often expressed indirectly (Example 2, "AC gak dingin"), and reviews are short, colloquial, and code-mixed, exactly the conditions under which document-level sentiment collapses distinct operational signals.

3.7. Dataset Split

Following the two-tier design, the experimental split leverages each tier for what it is best suited to: the **training set** comprises the 2,480 silver (LLM-labelled) reviews outside the gold subset; the **validation set** comprises 100 gold reviews; and the **test set** comprises the remaining 400 gold reviews. All reported test results are therefore computed exclusively on adjudicated, human-verified labels, while the larger silver tier provides the supervision signal for training. Table 8 consolidates the corpus partitions, their label provenance, and release conditions.

4. Methodology

4.1. Pipeline Overview

Figure 2 gives an end-to-end overview of the IndoHotelABSA methodology, from data construction to CRM application. The pipeline has four phases. *Data construction* (stages 1–3, Section 3) crawls Indonesian hotel reviews from the Google Places API across 55 cities, applies deduplication, length filtering, and privacy-preserving field removal, and draws a class-balanced 3,000-review sample. *Annotation* (stages 4–5, Section 3.4) pre-annotates each sampled review with an LLM to produce *silver* drafts, which three independent annotators then verify and correct into a *gold* standard (Fleiss' $\kappa = 0.894$). *Modelling and evaluation* (stages 6–9) splits the data into a 2,480-review silver training set and 100/400 gold validation/test sets, fine-tunes the encoders and HI-ABSA (and prompts/LoRA-tunes the LLMs), and evaluates on the gold test set with paired-bootstrap and McNemar significance testing. Finally, *application* (stage 10, Section 7) operationalises the aspect-level predictions into an explainable CRM dashboard and, with our prior cancellation-risk model, a dual-signal ICDA view. The remainder of this

Table 7
Representative gold-annotated reviews from IndoHotelABSA (verbatim, with English glosses). Each review maps to a set of (aspect, polarity) pairs over the seven CRM aspects.

#	Review (Indonesian, verbatim) / English gloss	Gold annotations
1	*"Haraga terlalu tinggi, tapi kamar cukup nyaman, makanan enak, wifi tersedia walau hanya pakai hotspot seluler, petugas sangat ramah, hanya harga untuk diving terlalu mahal."* "The price is too high, but the room is quite comfortable, the food is good, wifi is available though only via mobile hotspot, the staff are very friendly; only the diving price is too expensive."	Price/Value: NEG; Room & Facilities: POS; Food & Beverage: POS; Service/Staff: POS; Public Facilities: NEU
2	*"AC gak dingin, kamar mandi bocor gak tau air apaan, sarapan cuma roti ama teh manis."* "The AC isn't cold, the bathroom leaks some unknown water, breakfast is just bread and sweet tea."	Room & Facilities: NEG; Food & Beverage: NEG
3	*"Penginapan dengan pelayanan yang ramah 24 jam, harga bersahabat, kamar nyaman, ac normal, chanel tv banyak, kamar mandi bersih, disediakan handuk dan sandal."* "A guesthouse with friendly 24-hour service, affordable price, comfortable room, working AC, many TV channels, a clean bathroom, towels and slippers provided."	Cleanliness: POS; Service/Staff: POS; Room & Facilities: POS; Price/Value: POS

Table 8
IndoHotelABSA composition, label provenance, and release conditions. All partitions are released; labels are explicitly flagged as LLM-drafted (silver) or human-verified (gold).

Partition	#	Label source	Release
Full corpus	14,988	unlabelled text	text only, PII removed
Annotation sample	3,000	(for labelling)	class-balanced draw
Silver training	2,480	LLM-drafted	released, flagged silver
Gold validation	100	human-verified	released, gold
Gold test	400	human-verified	released, gold
Gold total	500	human-verified	1,506 aspect labels

section formalises the task, the proposed model, the baselines, and the evaluation protocol.

4.2. Task Formulation

Given a review r, the model predicts a set of (aspect, polarity) pairs. We decompose this into two sub-tasks and one joint measure: *Aspect Category Detection* (ACD), a multi-label decision over the seven aspects; *Aspect Category Polarity* (ACP), a three-way sentiment classification for each detected aspect; and *Joint ABSA*, in which a prediction is correct only when both the aspect and its polarity match the gold label. Equivalently, each review is represented as a vector of seven categorical labels, one per aspect, drawn from $\{\text{N/A}, \text{POS}, \text{NEG}, \text{NEU}\}$, where N/A denotes a non-mentioned aspect.

4.3. Proposed Model: HI-ABSA

Standard fine-tuning attaches seven classification heads to a single shared `[CLS]` representation, forcing all aspects to be decided from one pooled vector. This is sub-optimal for ABSA, where different aspects are evidenced by different tokens, and it yields no per-aspect interpretability. We propose **HI-ABSA** (Hospitality-Informed ABSA), an aspect-aware architecture that extends an Indonesian encoder with two components: *Aspect-Query Attention* (AQA), which pools aspect-specific evidence, and *Lexicon-Gated Fusion* (LGF), which injects a hospitality sentiment prior for low-resource robustness. HI-ABSA is the textual analogue of the domain-informed attention used in our structured-data churn model (Sika et al., 2026), giving the two papers a coherent design philosophy. Figure 3 depicts the architecture.

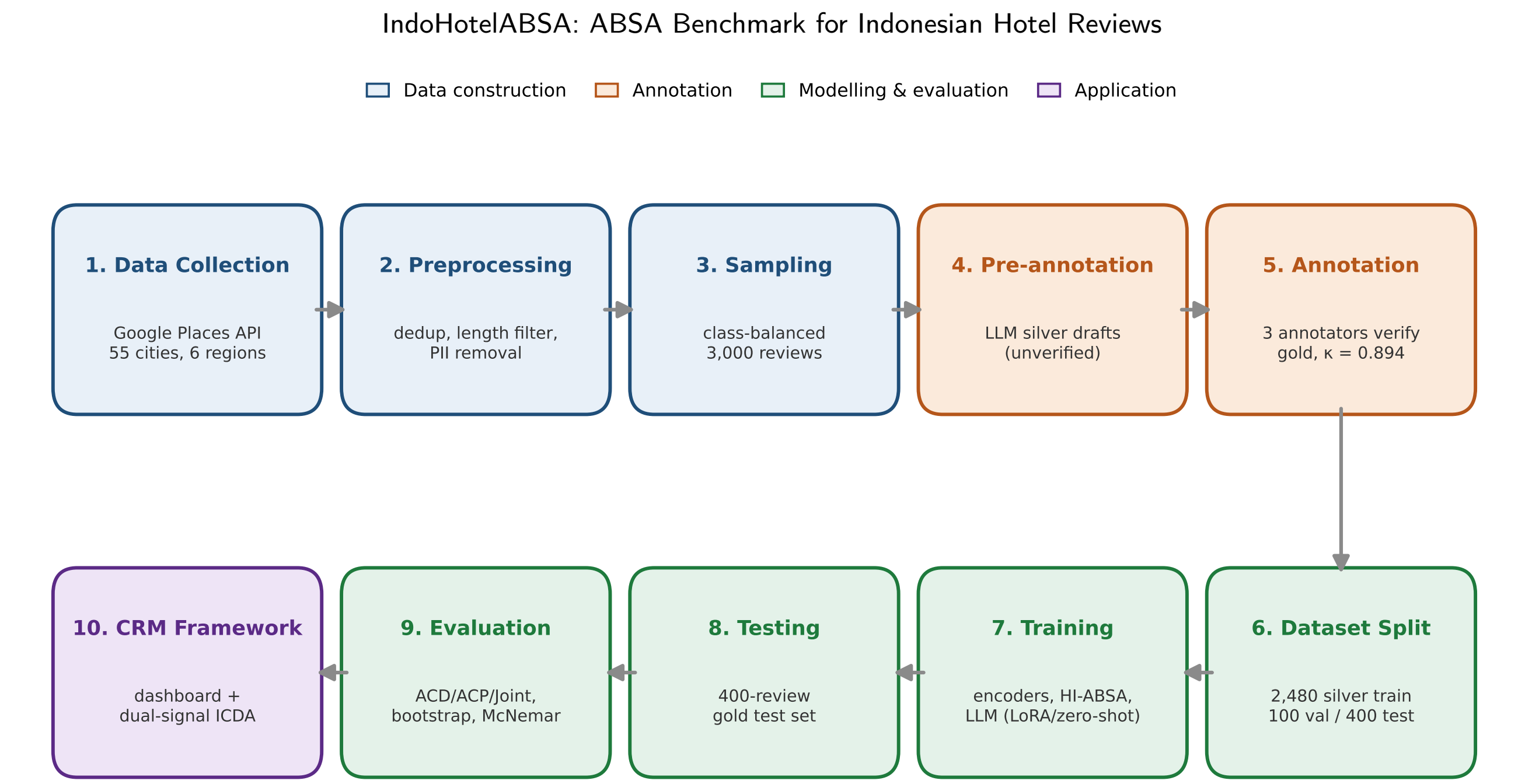

Figure 2: End-to-end IndoHotelABSA pipeline: data construction (crawling, preprocessing, sampling), annotation (LLM silver pre-annotation then human-verified gold), modelling and evaluation (split, training, testing, statistical evaluation), and CRM application (dashboard and dual-signal ICDA view).

Encoder. An input review x is encoded into contextual token embeddings

$$\mathbf{H} = \text{IndoBERT}(x) = [\mathbf{h}_1; \dots; \mathbf{h}_L] \in \mathbb{R}^{L\times d}. \tag{1}$$

Aspect-Query Attention (AQA). For each of the A=7 aspects we introduce a learnable query $\mathbf{q}_a \in \mathbb{R}^d$ that attends over the token sequence, pooling the evidence relevant to that aspect. Writing the scaled dot-product score between aspect query a and token i as $e_{a,i}$, the attention weights and pooled context are

$$e_{a,i} = \frac{1}{\sqrt{d}} (\mathbf{q}_a \mathbf{W}_Q)(\mathbf{h}_i \mathbf{W}_K)^\top, \tag{2}$$

$$\boldsymbol{\alpha}_a = \text{softmax}_i(e_{a,i}), \tag{3}$$

$$\mathbf{c}_a = \sum_{i=1}^{L} \alpha_{a,i} (\mathbf{h}_i \mathbf{W}_V) \in \mathbb{R}^d. \tag{4}$$

Each aspect thus obtains its own attention-pooled context vector $\mathbf{c}_a$, and the weights $\boldsymbol{\alpha}_a$ are directly interpretable as which tokens drove the model's decision for aspect a, feeding the CRM dashboard of Section 7.

Lexicon-Gated Fusion (LGF). In a low-resource setting, a domain sentiment lexicon is a valuable prior. For aspect a we compute a lexicon signal $\boldsymbol{\ell}_a = [\ell_a^+, \ell_a^-]$ from the counts of positive/negative hospitality-lexicon terms co-occurring with aspect a's cue words, and fuse it through a learnable gate:

$$g_a = \sigma\big(\mathbf{w}_g^\top [\mathbf{c}_a; \boldsymbol{\ell}_a] + b_g\big) \in (0, 1), \tag{5}$$

$$\tilde{\mathbf{c}}_a = \mathbf{c}_a + g_a (\mathbf{W}_\ell \boldsymbol{\ell}_a). \tag{6}$$

The gate g_a lets the model rely on the lexicon prior when the encoder signal is weak (rare aspects, short reviews) and suppress it otherwise.

Classification and training. Each fused representation is classified over the four labels {N/A, POS, NEG, NEU}:

$$\hat{\mathbf{p}}_a = \text{softmax}(\mathbf{W}_a \tilde{\mathbf{c}}_a + \mathbf{b}_a). \tag{7}$$

The model is trained end-to-end with a class-weighted cross-entropy summed over the seven aspects, $\mathcal{L} = \sum_{a=1}^{A} \sum_k w_k \, y_{a,k} \log \hat{p}_{a,k}$, where the weights w_k down-weight the dominant N/A class. We ablate each novel component (AQA, LGF) to quantify its contribution. Algorithm 1 summarises the full HI-ABSA forward pass.

4.4. Baseline Models

We compare HI-ABSA against five families of methods spanning the classical–neural–generative–LLM spectrum:

- **Lexicon baseline.** A rule-based method combining aspect keyword matching with an Indonesian sentiment lexicon, serving as a training-free lower bound.
- **Indonesian encoders.** IndoBERT (Wilie et al., 2020) and its social-media variant, fine-tuned with a shared encoder and seven four-way classification heads (one per aspect).
- **Multilingual encoders.** mBERT (Devlin et al., 2019) and XLM-RoBERTa (Conneau et al., 2020), fine-tuned identically, to test whether Indonesian-specific pre-training is necessary.

Algorithm 1 HI-ABSA Forward Pass

Require: Review x; aspect queries $\{\mathbf{q}_a\}_{a=1}^{A}$; hospitality lexicon
Ensure: Per-aspect predictions $\{\hat{\mathbf{p}}_a\}$; attention $\{\boldsymbol{\alpha}_a\}$

$\mathbf{H} \leftarrow \text{IndoBERT}(x)$ ▷ token embeddings, Eq. (1)
for $a \leftarrow 1$ to A **do** ▷ A=7 aspects
  $e_{a,i} \leftarrow (\mathbf{q}_a \mathbf{W}_Q)(\mathbf{h}_i \mathbf{W}_K)^\top / \sqrt{d}$ ▷ scores, Eq. (2)
  $\boldsymbol{\alpha}_a \leftarrow \text{softmax}_i(e_{a,i})$ ▷ attention, Eq. (3)
  $\mathbf{c}_a \leftarrow \sum_i \alpha_{a,i}(\mathbf{h}_i \mathbf{W}_V)$ ▷ aspect context, Eq. (4)
  $\boldsymbol{\ell}_a \leftarrow \text{LexiconCount}(x, a)$ ▷ $[\ell_a^+, \ell_a^-]$
  $g_a \leftarrow \sigma(\mathbf{w}_g^\top [\mathbf{c}_a; \boldsymbol{\ell}_a] + b_g)$ ▷ gate, Eq. (5)
  $\tilde{\mathbf{c}}_a \leftarrow \mathbf{c}_a + g_a(\mathbf{W}_\ell \boldsymbol{\ell}_a)$ ▷ fusion, Eq. (6)
  $\hat{\mathbf{p}}_a \leftarrow \text{softmax}(\mathbf{W}_a \tilde{\mathbf{c}}_a + \mathbf{b}_a)$ ▷ classify, Eq. (7)
return $\{\hat{\mathbf{p}}_a\}$, $\{\boldsymbol{\alpha}_a\}$

- **Generative seq2seq.** mT5 (Xue et al., 2021), a multilingual text-to-text model fine-tuned to generate the (aspect, polarity) set as a JSON string, testing whether a generative formulation is competitive with discriminative encoders in this low-resource setting.
- **Large language models.** An open LLM in two regimes: parameter-efficient fine-tuning with LoRA (Hu et al., 2022) (optionally 4-bit QLoRA (Dettmers et al., 2023)) and few-shot / zero-shot prompting (Brown et al., 2020; Touvron et al., 2023), to assess whether task-specific training is required for low-resource ABSA.

4.5. Implementation Details

Encoder models use a maximum sequence length of 192 tokens, AdamW with a learning rate of 2×10^{-5}, batch size 16, and early stopping on validation loss. Because the N/A class dominates (most reviews mention only a few aspects), the training loss down-weights N/A relative to the sentiment classes. For the few-shot LLM, prompts include a small number of balanced in-context examples and constrain the output to valid JSON over the fixed schema; malformed outputs are parsed leniently and, if unrecoverable, treated as empty. All experiments fix the random seed (42) for reproducibility. Table 9 lists the full training configuration and the software/hardware environment. To make the study fully auditable and in line with the journal's FAIR-data emphasis, Table 10 lists every released artifact and its status.

4.6. Evaluation Metrics

We report, for ACD, per-aspect precision/recall/F1 together with macro- and micro-averaged F1; for ACP, polarity accuracy and macro-F1 computed over correctly detected aspects; and, for Joint ABSA, precision/recall/F1 over (aspect, polarity) pairs. Per-aspect reporting is emphasised because CRM value depends on reliable detection of *each* operational domain, not only aggregate accuracy. Significance between the best model and baselines is assessed with bootstrap confidence intervals over the test set.

Table 9
Training hyperparameters and the software/hardware environment. Encoder settings are shared by all fine-tuned encoders and by HI-ABSA.

Setting	Value
Encoders / HI-ABSA (fine-tuning)	
Backbone encoder	IndoBERT-base-p1 (`indobenchmark`)
Max sequence length	192 tokens
Optimiser	AdamW
Learning rate	2×10^{-5}
Batch size	16
Epochs	up to 30, early stopping (patience 5)
Dropout	0.1
Loss	class-weighted cross-entropy (down-weight N/A)
Aspect queries (AQA)	7
Random seed	42
LLM (LoRA fine-tuning)	
Base model	Qwen2.5-0.5B-Instruct
LoRA r/α/dropout	16 / 32 / 0.05
LoRA target modules	q, k, v, o projections
Epochs / LR / batch	3 / 2×10^{-4} / 4 (fp16)
LLM (zero-shot)	Gemini Flash (commercial, no training)
Environment	
Language/framework	Python 3.14; PyTorch 2.11 (CUDA 12.8)
Libraries	transformers 4.57; peft 0.19
GPU	NVIDIA GeForce RTX 4050 Laptop

5. Experiments and Results

5.1. Experimental Setup

All models are trained on the 2,480-review silver set, model-selected on the 100-review gold validation set, and evaluated on the 400-review human-verified gold test set (Section 3.7) with the metrics of Section 4.6. Encoders and HI-ABSA are fine-tuned with the configuration of Section 4.5 using a fixed random seed (42). The zero-shot LLM row reports the raw predictions of the same commercial instruction-tuned LLM (Google Gemini Flash) used for silver pre-annotation, evaluated on the gold test set; the exact model version and access date are logged in the released configuration for reproducibility, as a commercial API is versioned and non-deterministic. Because the gold labels originate from human corrections of this model's drafts, its score carries a favourable anchoring bias, which we disclose and discuss in Section 6. To provide an *anchoring-free* reference point for the LLM paradigm, we additionally report a small open LLM (Qwen2.5-0.5B) LoRA-fine-tuned on the silver training set: it never produced any gold draft and is therefore free of anchoring, yet it underperforms the encoders, which indicates that the zero-shot advantage stems from scale

Table 10
Reproducibility and release checklist. All artifacts are released on acceptance (Mendeley Data and a public code repository).

Artifact	Content	Status
Corpus	14,988 reviews (text, rating, hotel, city)	Released
Gold labels	500 reviews, 1,506 aspect annotations	Released
Silver labels	2,480 LLM-drafted, flagged	Released
Split files	train/val/test membership	Released
Annotation guide	7-aspect schema + decision rules	Released
Model & baselines	HI-ABSA, encoders, LLM, lexicon	Released
Fine-tuning config	hyperparameters, seed 42	Reported
Evaluation scripts	metrics, bootstrap, McNemar/Holm	Released
HoASA cross-eval	SOTA-validation script	Released
Data statement	shared vs. restricted items	In submission

and the zero-shot regime rather than being an artefact of the anchoring bias alone.

5.2. Main Results

Because IndoHotelABSA is a newly released benchmark, no prior published numbers exist on it; we therefore compare against the state-of-the-art *method families* for this task, the Indonesian encoder IndoBERT (the strongest reported model on Indonesian NLU (Wilie et al., 2020)), multilingual encoders (mBERT, XLM-RoBERTa), instruction-tuned LLMs (zero-shot and LoRA), and a lexicon baseline, so that Table 12 constitutes a state-of-the-art comparison on the new corpus; we additionally validate our pipeline against the externally-published HoASA leaderboard in Section 5.8. The comparison yields three findings. *First*, Indonesian-specific pre-training matters decisively: the IndoBERT-based models (Joint-F1 0.751–0.758) outperform the multilingual encoders mBERT and XLM-RoBERTa (both 0.670) by more than eight points, and IndoBERTweet's social-media pre-training does not close the gap (0.710). *Second*, all neural models clearly surpass the lexicon baseline (0.428), whose polarity accuracy (0.526) confirms that rule-based matching cannot resolve clause-level sentiment; the generative seq2seq model mT5 (0.668) is competitive with the multilingual encoders but remains below the Indonesian encoders, indicating that a generative formulation does not by itself overcome the low-resource limitation. *Third*, and most strikingly, LLM performance is governed by scale and regime, not merely by being an LLM: the *zero-shot* large LLM attains 0.872 Joint-F1, exceeding the best fine-tuned encoder by 11.4 points, whereas a small LLM (Qwen2.5-0.5B) *LoRA-fine-tuned* on the same silver data reaches only 0.684, below every encoder. In other words, the LLM advantage comes from scale and zero-shot capability, not from fine-tuning a compact LLM. HI-ABSA matches the strongest fine-tuned baseline (0.751 vs. 0.756, difference not significant) while uniquely providing per-aspect attention interpretability. The precision/recall split in Table 12 further characterises the systems: HI-ABSA and IndoBERT are recall-oriented (Joint-R > Joint-P), reflecting a tendency to over-predict aspects, whereas the LoRA LLM is precision-oriented (0.794 vs. 0.600), conservatively under-predicting, and the zero-shot LLM is balanced and high on both (0.890/0.855). The zero-shot figure must be read together with the anchoring caveat below.

Statistical significance. Paired bootstrap tests (1,000 resamples of the 400-review test set) on Joint-F1 show that the difference between HI-ABSA and IndoBERT is not significant (-0.005, 95% CI $[-0.019, +0.007]$, $p = 0.44$), i.e. the two are statistically comparable. This equivalence is robust to random initialisation: over five seeds, IndoBERT and HI-ABSA reach mean Joint-F1 of 0.761 ± 0.005 and 0.764 ± 0.004 respectively (the single-seed figures in Table 12 lie within this variance), with HI-ABSA showing marginally higher mean and lower variance. In contrast, both LLM-related gaps are large and significant: the zero-shot LLM over the best encoder (+0.114, 95% CI $[+0.092, +0.137]$, $p < 0.001$), and the encoder over the LoRA-tuned small LLM (+0.073, 95% CI $[+0.045, +0.101]$, $p < 0.001$). Table 11 extends this analysis with McNemar paired tests of HI-ABSA against *every* baseline on per-cell (review, aspect) correctness, with Holm correction for the eight comparisons. The pattern is clear: HI-ABSA is statistically indistinguishable from the strongest encoder (IndoBERT, $p = 0.38$) while *significantly* outperforming both multilingual encoders, IndoBERTweet, the lexicon baseline, and the LoRA-tuned small LLM (all Holm $p < 0.001$); only the anchoring-biased zero-shot LLM is significantly stronger. We therefore frame HI-ABSA not as a state-of-the-art accuracy claim but as an *operationally credible, CRM-preferred alternative*: statistically on par with the best locally-deployable model while uniquely providing native per-aspect interpretability (Section 7). Consistent with this, the paper's primary contributions are the benchmark and the explainable CRM framework, not a claim of a superior neural architecture.

5.3. Polarity-Class Breakdown

Aggregate polarity accuracy conceals a systematic weakness. Table 13 decomposes polarity performance into per-class F1 and reveals that every *fine-tuned* model essentially fails on the NEUTRAL class (F1 0.04–0.12), while performing strongly on POSITIVE and NEGATIVE (0.88–0.91). This is why polarity *macro*-F1 (ACP-mF1 in Table 12) is far below polarity accuracy for all encoders: accuracy is dominated by the two frequent classes, whereas macro-F1 exposes the neutral collapse. The cause is class rarity: only 69 of 1,506 gold annotations (4.6%) are neutral (Section 3.5), too few for the encoders to learn a reliable decision boundary. Strikingly, the only model that handles neutrals materially better is the zero-shot large LLM (F1 0.48), whose broad pre-training supplies a neutral prior that supervised low-resource training cannot. We report this openly: neutral sentiment

Table 11
McNemar paired tests of HI-ABSA (proposed) against each baseline on per-cell (review, aspect) correctness (400-review gold test set), with Holm correction. b = HI-ABSA correct / baseline wrong; c = HI-ABSA wrong / baseline correct.

HI-ABSA vs.	b	c	χ^2	p (Holm)
Lexicon baseline	625	179	246.3	< 0.001
mBERT	322	88	132.4	< 0.001
XLM-RoBERTa	388	96	175.0	< 0.001
IndoBERTweet	206	92	42.9	< 0.001
IndoBERT	73	85	0.8	0.38
mT5-small (generative)	300	168	36.7	< 0.001
LLM LoRA	299	209	15.6	< 0.001
LLM zero-shot†	76	339	165.4	< 0.001

†Significant *in favour of* the (anchoring-biased) zero-shot LLM ($c > b$); all other significant rows favour HI-ABSA.

is the principal unsolved sub-problem of Indonesian hotel ABSA and a target for future work (e.g., neutral-focused augmentation or thresholding), not a detail hidden by headline accuracy. The confusion matrix in Figure 5 makes this concrete: of the 55 gold neutral cells HI-ABSA recovers only one (2% recall), misclassifying 33 as positive and 18 as negative, whereas positive and negative aspects are recovered reliably (84% and 80% recall). The off-diagonal mass also shows the model's mild tendency to over-predict aspects (N/A misread as positive or negative), consistent with the recall-oriented error profile of Section 5.7.

5.4. Ablation Study

Table 14 isolates the contribution of the two novel components. We report this ablation with full transparency: all four variants fall within a one-point band (0.749–0.758), and a paired bootstrap between the full model and the shared-`[CLS]` variant confirms the difference is not significant (-0.006, 95% CI $[-0.017, +0.007]$, $p = 0.40$). In other words, on this dataset *aspect-query attention and the lexicon prior neither help nor hurt joint accuracy*. The practical value of AQA is therefore not accuracy but *interpretability*: it yields per-aspect token attention (Eq. 3) that the shared-`[CLS]` architecture cannot produce, at no measurable accuracy cost, mirroring the accuracy-parity-plus-native-explainability positioning of our structured-data churn model (Sika et al., 2026). We conjecture that the silver training labels bound what any of these variants can learn (see Section 6).

5.5. Per-Aspect Analysis

Table 15 breaks down HI-ABSA detection (ACD) F1 by aspect. The explicitly-cued commercial and experiential aspects are easiest, Service/Staff (0.922), Room & Facilities (0.912), and Price/Value (0.908), because they are signalled by frequent, unambiguous keywords (*ramah*, *kamar*, *harga*). The hardest is Public Facilities (0.767), whose surface forms are diverse (Wi-Fi, parking, pool, lift) and frequently implicit, followed by Cleanliness (0.793), which is often expressed indirectly (odour, pests, stains) rather than with the word *bersih*. This pattern is consistent across all encoder models and indicates that residual errors concentrate on lexically-diffuse and implicit aspects rather than on any systematic model deficiency.

5.6. Efficiency and Cost

The two competitive approaches occupy opposite points on the cost spectrum. The fine-tuned encoders (IndoBERT, HI-ABSA) require one-time training but then run locally at negligible marginal cost and with full data privacy, important for hotel operators handling guest data. The zero-shot LLM needs no training but incurs a per-call API cost and sends review text to a third-party service at inference time. For high-volume, privacy-sensitive CRM deployment, a locally hosted encoder is therefore attractive despite its accuracy gap, whereas the LLM is preferable when annotation budget is the binding constraint. This trade-off, rather than raw accuracy alone, should guide method selection in practice.

5.7. Interpretability and Error Analysis

A per-error-type breakdown clarifies where the two leading systems differ. For HI-ABSA, 47% of cell errors are spurious aspects (false positives, concentrated in Room & Facilities and Cleanliness), 30% are polarity confusions, and 23% are missed aspects; the dominant polarity confusion is negative→positive (55 cases), typical of negated or mixed-sentiment clauses (*"kamarnya bagus tapi kotor"*). The zero-shot LLM makes far fewer errors overall (221 vs. 484 cells) and errs in the opposite direction: it is conservative, with 42% of its errors being missed aspects (chiefly Cleanliness) rather than spurious ones, consistent with high precision. Across both systems, the recurring difficulties are implicit aspects, negation, and code-mixing, the phenomena that motivate a dedicated Indonesian hospitality resource. Figure 7 contrasts the error profiles. As a *supplementary*, per-prediction explanation, the Aspect-Query Attention weights of HI-ABSA (Eq. 3) expose the tokens that drove each aspect decision. We assess their faithfulness quantitatively rather than by cherry-picked examples: across 614 correctly-predicted aspect cells, the top-attended token is a content word (not a stopword or punctuation) in 61.7% of cases, so the attention offers useful but imperfect token-level evidence. We therefore treat it as an optional drill-down beneath the aggregate CRM dashboard of Section 7, which, being aggregated and auditable, remains the primary interpretability artifact. Table 16 illustrates these patterns with representative correct and incorrect HI-ABSA predictions.

5.8. Cross-Benchmark Validation on HoASA (SOTA Comparison)

Because IndoHotelABSA is new, Table 12 compares method families *on our corpus* but cannot be checked against externally published numbers. To provide a genuine state-of-the-art comparison on an *established* benchmark, we additionally train our two encoders on **HoASA**, the hotel ABSA dataset of the IndoNLU benchmark (Wilie et al., 2020) (ten aspects, four-way labels; 2,283/285/286 train/dev/test), under the same fine-tuning protocol and evaluate with the

Table 12
Main results on the 400-review human-verified gold test set. ACD-F1 = aspect-category detection macro-F1; ACP-Acc / ACP-mF1 = aspect-category polarity accuracy / macro-F1 over the three polarity classes; Joint-P/R/F1 = precision/recall/F1 over (aspect, polarity) pairs. Column-best in **bold**; best among locally-deployable models underlined. †Same LLM used for silver pre-annotation; score carries a favourable anchoring bias (Section 6).

Model	ACD-F1	ACP-Acc	ACP-mF1	Joint-P	Joint-R	Joint-F1
Lexicon baseline	0.794	0.526	0.465	0.422	0.434	0.428
mBERT	0.771	0.840	0.586	0.608	0.747	0.670
XLM-RoBERTa	0.760	0.859	0.598	0.586	0.782	0.670
IndoBERTweet	0.802	0.858	0.599	0.668	0.757	0.710
IndoBERT	0.850	<u>0.872</u>	<u>0.630</u>	<u>0.731</u>	0.784	<u>0.756</u>
HI-ABSA (ours)	<u>0.853</u>	0.866	0.604	0.718	<u>0.787</u>	0.751
mT5-small (generative)	0.770	0.837	0.600	0.702	0.638	0.668
LLM LoRA (Qwen2.5-0.5B)	0.745	0.881	0.643	0.794	0.600	0.684
LLM zero-shot (Gemini)†	**0.933**	**0.926**	**0.793**	**0.890**	**0.855**	**0.872**

Table 13
Per-polarity F1 over correctly detected aspects (gold test set), with polarity macro-F1. All fine-tuned models collapse on the rare NEU class; only the zero-shot LLM handles it. †anchoring bias (Section 6).

Model	F1-POS	F1-NEG	F1-NEU	ACP-mF1
IndoBERT	0.882	0.904	0.105	0.630
HI-ABSA (ours)	0.877	0.898	0.036	0.604
LLM LoRA (Qwen2.5-0.5B)	0.909	0.901	0.120	0.643
LLM zero-shot (Gemini)†	**0.943**	**0.953**	**0.481**	**0.793**

Table 14
Ablation of HI-ABSA components on the gold test set (Δ vs. full model). All differences lie within bootstrap noise.

Variant	Joint-F1	Δ
Full HI-ABSA	0.751	–
w/o AQA (shared `[CLS]`)	0.758	+0.007
w/o LGF (no lexicon)	0.749	−0.002
w/o AQA & LGF (encoder+heads)	0.757	+0.006

official IndoNLU metric (macro-F1 over the flattened aspect-cells). Table 17 places our results beside the published IndoNLU leaderboard. Two observations follow. *First*, our IndoBERT fine-tuning reproduces the published IndoBERT-base result almost exactly (0.9216 vs. 0.9207), validating that our experimental pipeline is correctly calibrated against the state of the art. *Second*, our base-size HI-ABSA is competitive on this external benchmark (0.9146, on par with XLM-R-base 0.9157), consistent with our central finding that aspect-query attention matches, rather than beats, a strong shared-encoder baseline; the highest published scores use *large* models (XLM-R-large 0.9427, IndoBERT-large 0.9375) outside our base-size regime. This cross-benchmark check confirms our methods sit within the established SOTA band on an independent, peer-reviewed dataset.

Table 15
Per-aspect detection (ACD) F1 of HI-ABSA on the gold test set.

Aspect	F1
Service/Staff	0.922
Room & Facilities	0.912
Price/Value	0.908
Food & Beverage	0.872
Location	0.800
Cleanliness	0.793
Public Facilities	0.767
Macro-F1	0.853

6. Discussion

6.1. Does Aspect-Aware Modelling Help? (RQ2)

The ablation (Table 14) shows that Aspect-Query Attention and Lexicon-Gated Fusion do not significantly change joint accuracy on this dataset. We interpret this as a *data-bound*, not an architecture-bound, result: with supervision drawn from silver (LLM) labels and a 400-review gold test, the ceiling that separates architectural variants is narrow, and a strong Indonesian encoder already captures most of the extractable aspect signal. The contribution of AQA is therefore best understood as *free interpretability*: it produces per-aspect attention distributions (Eq. 3) that a shared-`[CLS]` head cannot, at no measurable accuracy cost. This mirrors the finding in our structured-data churn model (Sika et al., 2026), where the proposed components match a strong baseline's accuracy while adding native, actionable explanations, the property that matters for CRM deployment. We refrain from over-claiming an accuracy benefit, and report the null result transparently.

6.2. Fine-Tuned Encoders vs. LLMs (RQ3)

The results paint a scale-dependent picture. A *zero-shot* large LLM substantially and significantly outperforms every fine-tuned encoder (0.872 vs. 0.756 Joint-F1, $p < 0.001$), yet a *LoRA-fine-tuned* small LLM (Qwen2.5-0.5B)

Table 16
Representative HI-ABSA predictions on the gold test set (reviews verbatim, shortened). Polarity: + positive, − negative, 0 neutral. Clean = Cleanliness, Serv = Service, Room = Room & Facilities, Loc = Location.

	Review (Indonesian, shortened)	Gold	HI-ABSA	Outcome
✓	*"AC gak dingin, kamar mandi bocor..., sarapan cuma roti ama teh manis"*	Room:−, Food:−	Room:−, Food:−	correct
✓	*"...pelayanan ramah 24 jam, harga bersahabat, kamar nyaman...kamar mandi bersih..."*	Clean:+, Serv:+, Room:+, Price:+	Clean:+, Serv:+, Room:+, Price:+	correct
×	*"PENGINAPAN TERBURUK...KARYAWAN...SANGAT TIDAK RAMAH"*	Serv:−	Serv:−, Room:−	spurious aspect (Room)
×	*"...tempat nya bersih dan nyaman, terutama abg yg disana baik2"*	Clean:+, Serv:+, Room:+	Clean:+, Room:+	missed implicit aspect (Serv)
×	*"...lokasi nya lumayan jauh dari jalan utama"*	Loc:−, Clean:−, Room:−	Loc:+, Clean:−, Room:−	polarity flip (Loc −→+)

Table 17
State-of-the-art comparison on the external HoASA benchmark (Wilie et al., 2020) (test-set F1, IndoNLU metric = macro-F1 over flattened aspect-cells). Published rows are from the IndoNLU leaderboard; *ours* are trained under our protocol. Best overall in **bold**; best base-size underlined.

Model	Size	HoASA F1
Scratch (IndoNLU)	–	0.7628
mBERT (IndoNLU)	base	0.8463
XLM-R (IndoNLU)	base	0.9157
IndoBERT-p1 (IndoNLU)	base	0.9207
IndoBERT-p1 (IndoNLU)	large	0.9375
XLM-R (IndoNLU)	large	**0.9427**
IndoBERT (ours)	base	0.9216
HI-ABSA (ours)	base	0.9146

significantly *under*performs the encoders (0.684, $p < 0.001$). The determinant is therefore model scale and the zero-shot regime, not the LLM paradigm per se: parameter-efficient fine-tuning of a compact LLM does not substitute for a purpose-built encoder, whereas a large instruction-tuned model succeeds without any task supervision. This nuances the reality-check of Zhang et al. (2024): in a *low-resource* setting with only silver supervision, the encoder-vs-LLM balance inverts for sufficiently large LLMs. Two caveats temper the zero-shot result. First, an *anchoring bias*: the gold labels are human corrections of this same LLM's drafts, so annotators may have retained the LLM's framing on ambiguous cells, inflating its apparent score; the 7.9% human-correction rate (Section 3.5) bounds but does not eliminate this effect, notably, the LoRA LLM, free of this bias, does not benefit. Second, cost and privacy (Section 5.6) favour local encoders. The practical takeaway is not that LLMs render encoders obsolete, but that method choice is governed by the scale, annotation-budget, cost, and privacy constraints of the deployment.

6.3. Practical Implications for Hospitality CRM (RQ4)

Regardless of which model produces them, aspect-level predictions become operational through the framework of Section 7: per-aspect net sentiment scores (Eq. 8) and severity ranking convert free-text reviews into a prioritised, interpretable dashboard. HI-ABSA's attention weights additionally trace each aspect decision back to specific tokens, so a revenue manager sees not only *that* cleanliness is trending negative but *which* complaints drive it. Coupled with the cancellation-risk model of our prior work (Sika et al., 2026), this yields the dual-signal view (Section 7.4): the churn model flags at-risk bookings, and the ABSA dashboard explains the experiential causes.

6.4. Positioning Relative to Prior Indonesian ABSA

Beyond model comparison, the study's durable contribution is the resource itself. Relative to HoASA (Table 1), IndoHotelABSA is an order of magnitude larger, spans 55 cities nationwide rather than a single platform, adopts a CRM-aligned seven-aspect schema, and is released with a documented LLM-assisted–human-verified protocol at almost-perfect agreement ($\kappa = 0.894$). The strong absolute scores of all neural models, and the clean per-aspect error structure, indicate that the corpus provides a reliable and challenging benchmark for future Indonesian ABSA research. A direct cross-benchmark check on HoASA (Section 5.8) confirms that our pipeline reproduces the published IndoNLU SOTA (IndoBERT 0.9216 vs. 0.9207) and that HI-ABSA is competitive on that external dataset.

7. Explainable CRM Framework

The value of aspect-level sentiment for hospitality CRM lies not in individual predictions but in *aggregated, actionable* views of the guest experience. Unlike post-hoc explanation methods such as SHAP (Lundberg and Lee, 2017), which attribute a scalar prediction after the fact, our HI-ABSA model

Table 18
Suitability for CRM deployment. ACD-F1 = aspect-detection macro-F1 (what the dashboard aggregates); Interp. = native per-aspect explanation; Local = on-premise/private; Free = no per-inference cost. HI-ABSA uniquely satisfies all CRM criteria.

Model	Joint-F1	ACD-F1	Interp.	Local	Free
IndoBERT	0.756	0.850	no	yes	yes
LLM zero-shot (Gemini)	**0.872**	**0.933**	no	no	no
**HI-ABSA (ours)**	0.751	**0.853***	**yes**	**yes**	**yes**

*Best aspect-detection F1 among locally-deployable models.

exposes per-aspect attention (Eq. 3) natively at inference time. This section describes how IndoHotelABSA predictions are operationalised into a CRM decision-support layer, and how this qualitative signal complements the quantitative cancellation-risk signal from our prior work (Sika et al., 2026).

7.1. Model Choice for CRM Deployment: Why HI-ABSA

Raw Joint-F1 is only one of several criteria that matter for a deployed CRM system; a hotel operator also needs *aspect-level* reliability (the dashboard aggregates per aspect), *native explanations* (managers must see *why* an aspect is flagged), *data privacy* (guest reviews are sensitive), and *bounded cost*. Table 18 evaluates the leading models on these CRM-oriented axes and shows that HI-ABSA, not the most accurate model overall, is the *CRM-preferred* choice. Among locally-deployable models, the only option when guest data cannot leave the premises, HI-ABSA attains the highest aspect-detection score (ACD-F1 0.853), which is precisely the quantity the aspect-aggregated dashboard consumes, while being the only model to expose native per-aspect attention (Eq. 3) at no accuracy cost relative to the best encoder. The zero-shot LLM is more accurate but sends every review to a third-party API, offers no per-aspect attribution, and incurs a recurring per-call cost, disqualifying properties on privacy or budget grounds. HI-ABSA thus occupies the CRM "sweet spot": competitive accuracy, best-in-class local aspect detection, native interpretability, on-premise privacy, and zero marginal inference cost.

7.2. From Aspect Predictions to a CRM Dashboard

For a set of reviews $\mathcal{R}$ scoped to a property and time window, the model yields per-review (aspect, polarity) pairs. For each aspect a we compute a *net sentiment score*

$$s_a = \frac{n_a^+ - n_a^-}{n_a^+ + n_a^- + n_a^0}, \quad (8)$$

where n_a^+, n_a^-, n_a^0 count positive, negative, and neutral mentions of aspect a in $\mathcal{R}$. The vector $\mathbf{s} = [s_a]_{a=1}^7$ forms a *CRM aspect dashboard*: a compact, interpretable summary of which operational domains (cleanliness, service, price, ...) are driving guest satisfaction or dissatisfaction for that property and period. Tracking $\mathbf{s}$ over consecutive windows surfaces emerging problems (e.g., a declining cleanliness score) before they manifest in headline ratings.

This aggregate dashboard, rather than any single-prediction explanation, is the *primary* interpretability artifact of our framework: because it aggregates many predictions, it is robust to the per-example noise inherent in token-level attention (Section 5.7), yet remains fully transparent, each score being a simple, auditable count ratio. Figure 8 instantiates it on the HI-ABSA predictions for the test set: panel (a) shows the per-aspect net sentiment s_a, and panel (b) ranks aspects by the severity signal of Section 7.3. The dashboard immediately localises the actionable domains, Cleanliness, Room & Facilities, and Service are net-negative and top the severity ranking, whereas Location is strongly positive ($s = +0.96$), directing managerial attention without any manual reading of individual reviews.

7.3. Aspect-Level Prioritisation

Because operational resources are limited, the dashboard ranks aspects by a severity signal combining net sentiment with mention volume, $\text{severity}_a = (1 - s_a) \cdot \log(1 + n_a^-)$, directing managerial attention to aspects that are both negative and frequently mentioned. Unlike a single star rating or a document-level sentiment score, this ranking is *directly actionable*: it names the operational domain to fix.

7.4. Integrated Customer Data Analysis (ICDA): Coupling Sentiment with Cancellation Risk

Hospitality CRM ultimately seeks to retain guests and revenue. We frame the two complementary signals as an *Integrated Customer Data Analysis* (ICDA) view of the guest, comprising a *predictive* pillar and a *perceptual* pillar. Our prior work (Sika et al., 2026) supplies the predictive pillar, predicting booking-cancellation risk from structured reservation data and exposing a group-level risk dashboard; the present work supplies the perceptual pillar, predicting aspect-level sentiment from unstructured reviews. Together they form the dual-signal ICDA view: the churn model identifies *who* and *how many* are at risk of leaving, while the ABSA model explains *why* guests are dissatisfied. Aligning the two, e.g., correlating a property's negative cleanliness or service scores with its cancellation rate, lets managers connect qualitative causes to quantitative outcomes and target interventions where they most affect retention. A full empirical coupling of the two signals on paired data is left to future work; here we establish the interpretable ABSA component and its dashboard interface.

7.5. Illustrative Use Case

Consider a city hotel whose overall rating is stable but whose dashboard shows a falling service score driven by a rising volume of negative Service/Staff mentions (slow check-in, unresponsive front desk). The severity ranking flags Service as the top priority; the manager can act (staffing, training) before the decline reaches the headline rating or inflates cancellations, an intervention that neither a star rating nor a document-level sentiment tool would have surfaced at the aspect level.

8. Limitations

Several limitations should be considered when interpreting these results. *(i) Data source.* Reviews are collected from a single mapping platform's public API; although coverage spans 55 cities, platform-specific reviewer demographics and moderation may introduce selection bias. *(ii) Annotation.* A class-balanced sample rather than the full corpus is annotated, and the LLM-assisted protocol, while human-verified, may leave residual bias from the initial machine drafts; we mitigate this with independent multi-annotator verification and report inter-annotator agreement. *(iii) Fixed aspect schema.* The seven CRM aspects are fixed a priori; implicit or emerging aspects outside this schema are not captured. *(iv) Language scope.* The corpus is monolingual Indonesian; heavy code-mixing and regional languages are only partially represented, and cross-lingual generalisation is not evaluated. *(v) Lexicon dependence.* The Lexicon-Gated Fusion component relies on a hospitality sentiment lexicon whose coverage bounds its contribution. *(vi) Dual-signal coupling.* The link between aspect sentiment and cancellation risk is formulated conceptually (Section 7.4); an empirical coupling on paired review–reservation data is left to future work. *(vii) Anchoring bias.* The headline zero-shot LLM score is inflated by an anchoring effect, as the gold labels are human corrections of that model's own drafts; we disclose this throughout and provide an anchoring-free LoRA reference, but the exact magnitude of the effect is not fully isolated. *(viii) Data licensing.* The corpus originates from a third-party platform's public API; although collected under its terms and released for non-commercial research, redistribution and reuse remain subject to that platform's terms, which users must observe.

9. Conclusion

This paper introduced **IndoHotelABSA**, a large-scale, nationwide benchmark for aspect-based sentiment analysis of Indonesian hotel reviews (14,988 reviews across 55 cities; a 500-review gold subset triply annotated at Fleiss' $\kappa = 0.894$), together with **HI-ABSA**, an aspect-aware model, and an explainable CRM framework. Three empirical findings stand out. First, Indonesian-specific pre-training is decisive: IndoBERT-based models exceed multilingual encoders by over eight Joint-F1 points. Second, LLM performance is scale-dependent: a zero-shot large LLM significantly outperforms all fine-tuned encoders (+0.114 Joint-F1, $p < 0.001$; tempered by an explicit anchoring caveat), whereas a LoRA-fine-tuned small LLM significantly underperforms them (−0.073), showing the advantage stems from scale and the zero-shot regime rather than the LLM paradigm itself. Third, the neutral class is the principal unsolved sub-problem: every fine-tuned model collapses on it, and only the large zero-shot LLM handles it materially better.

Crucially, the value of this work does not rest on topping an accuracy leaderboard. Its primary contribution is the benchmark resource itself, together with these findings and an explainable CRM framework. Within that framework we deliberately position HI-ABSA not as a state-of-the-art accuracy claim but as an *operationally credible, CRM-preferred alternative*: it is statistically indistinguishable from the strongest locally-deployable encoder ($p = 0.44$; corroborated by McNemar, $p = 0.38$) while uniquely adding native per-aspect interpretability, on-premise privacy, and zero per-inference cost, precisely the properties a real hotel CRM deployment requires but that the more accurate, third-party zero-shot LLM lacks. A cross-benchmark check on the external HoASA leaderboard confirms our pipeline reproduces the published IndoNLU state of the art (0.9216 vs. 0.9207). By coupling the resulting qualitative signal with our prior cancellation-risk model (Sika et al., 2026), the work advances a dual-signal Integrated Customer Data Analysis (ICDA) view of hospitality CRM, to which this paper contributes the perceptual (sentiment) pillar. Future work includes an empirical dual-signal coupling on paired review–reservation data, neutral-focused methods (targeted augmentation or thresholding) to resolve the neutral collapse, adaptive aspect discovery, and extension to regional Indonesian languages.

CRediT authorship contribution statement

Xaverius Sika: Conceptualization, Methodology, Software, Investigation, Data curation, Writing – original draft. **Eko Sediyono:** Conceptualization, Supervision, Writing – review & editing. **Irwan Sembiring:** Supervision, Validation, Writing – review & editing. **Teguh Wahyono:** Supervision, Validation, Writing – review & editing. **Eko Arip Winanto:** Software, Validation, Writing – review & editing.

Declaration of competing interest

The authors declare that they have no known competing financial interests or personal relationships that could have appeared to influence the work reported in this paper.

Declaration of generative AI and AI-assisted technologies in the manuscript preparation process

During the preparation of this work the authors used a large language model (Google Gemini) in order to produce *draft* (silver) aspect–polarity annotations for the IndoHotelABSA corpus. Every draft label was subsequently reviewed and, where necessary, corrected by three independent human annotators; all reported test evaluations use the human-verified gold labels. The language model was not used to generate the scientific content, analysis, or text of this manuscript. After using this tool, the authors reviewed and edited the content as needed and take full responsibility for the content of the published article.

Funding

This research was supported by Universitas Dinamika Bangsa (UNAMA), Jambi, Indonesia.

Data and code availability

The IndoHotelABSA corpus and annotation guidelines are deposited on Mendeley Data (reserved DOI: 10.17632/9vhzg5wkf9.1) under a CC BY 4.0 licence. The implementation code is available for peer review at an anonymized mirror (https://anonymous.4open.science/r/IndoHotelABSA-6BB8); the public GitHub release under an MIT licence will be linked on acceptance. Both the dataset and the code will be made public upon acceptance, with a companion data descriptor submitted separately to *Data in Brief*. The released corpus distinguishes LLM-drafted (silver) from human-verified (gold) labels, and all reported test evaluations use the human-verified gold labels. *For peer review*, the draft record can be accessed through the private preview link https://data.mendeley.com/preview/9vhzg5wkf9?a=45d46b9e-e351-413d-bb5c-119c670f8d48, which will be replaced by the public DOI on publication.

Acknowledgements

The authors thank the three annotators for their careful, independent verification of the IndoHotelABSA gold set, and Universitas Dinamika Bangsa (UNAMA), Jambi, and Satya Wacana Christian University (UKSW), Salatiga, Indonesia, for supporting this research.

References

Al Saidat, M.R., Shaalan, K., Yerima, S.Y., 2026. A hybrid deep learning framework for Arabic smishing detection: Dataset creation, model design, and explainability. Natural Language Processing Journal 16, 100222. doi:10.1016/j.nlp.2026.100222.

Birjali, M., Kasri, M., Beni-Hssane, A., 2021. A comprehensive survey on sentiment analysis: Approaches, challenges and trends. Knowledge-Based Systems 226, 107134. doi:10.1016/j.knosys.2021.107134.

Brown, T.B., Mann, B., Ryder, N., Subbiah, M., et al., 2020. Language models are few-shot learners, in: Advances in Neural Information Processing Systems (NeurIPS), pp. 1877–1901.

Cahyawijaya, S., Lovenia, H., Aji, A.F., Winata, G.I., et al., 2023. NusaCrowd: Open source initiative for Indonesian NLP resources, in: Findings of the Association for Computational Linguistics: ACL 2023, pp. 13745–13818.

Cahyawijaya, S., Winata, G.I., Wilie, B., et al., 2021. IndoNLG: Benchmark and resources for evaluating Indonesian natural language generation, in: Proceedings of EMNLP 2021, pp. 8875–8898.

Cohen, J., 1960. A coefficient of agreement for nominal scales. Educational and Psychological Measurement 20, 37–46.

Conneau, A., Khandelwal, K., Goyal, N., Chaudhary, V., Wenzek, G., Guzmán, F., Grave, E., Ott, M., Zettlemoyer, L., Stoyanov, V., 2020. Unsupervised cross-lingual representation learning at scale, in: Proceedings of ACL 2020, pp. 8440–8451.

Dettmers, T., Pagnoni, A., Holtzman, A., Zettlemoyer, L., 2023. QLoRA: Efficient finetuning of quantized LLMs, in: Advances in Neural Information Processing Systems (NeurIPS).

Devlin, J., Chang, M.W., Lee, K., Toutanova, K., 2019. BERT: Pre-training of deep bidirectional transformers for language understanding, in: Proceedings of NAACL-HLT 2019, pp. 4171–4186.

Dharshini, B.S., Mahendran, R.K., 2026. SENTICARE-GPT: A cross-fused generative transformer for emotion-aware and explainable sentiment analysis of patient feedback. Natural Language Processing Journal doi:10.1016/j.nlp.2026.100227. in press, article 100227.

Gebru, T., Morgenstern, J., Vecchione, B., Vaughan, J.W., Wallach, H., Daumé III, H., Crawford, K., 2021. Datasheets for datasets. Communications of the ACM 64, 86–92. doi:10.1145/3458723.

Guo, Y., Barnes, S.J., Jia, Q., 2017. Mining meaning from online ratings and reviews: Tourist satisfaction analysis using latent Dirichlet allocation. Tourism Management 59, 467–483. doi:10.1016/j.tourman.2016.09.009.

Hoang, M., Bihorac, O.A., Rouces, J., 2019. Aspect-based sentiment analysis using BERT, in: Proceedings of the 22nd Nordic Conference on Computational Linguistics (NoDaLiDa), pp. 187–196.

Hu, E.J., Shen, Y., Wallis, P., Allen-Zhu, Z., Li, Y., Wang, S., Wang, L., Chen, W., 2022. LoRA: Low-rank adaptation of large language models, in: International Conference on Learning Representations (ICLR).

Islam, A., Hasan, M.M., Morol, M.K., Fahad, N., Hosain, M.T., Hossen, M.J., Nandi, D., 2026. BAN-ASTE: A unified neural framework for Bengali aspect sentiment triplet extraction. Natural Language Processing Journal 16, 100216. doi:10.1016/j.nlp.2026.100216.

Koto, F., Lau, J.H., Baldwin, T., 2021. IndoBERTweet: A pretrained language model for Indonesian Twitter with effective domain-specific vocabulary initialization, in: Proceedings of EMNLP 2021, pp. 10660–10668.

Koto, F., Rahimi, A., Lau, J.H., Baldwin, T., 2020. IndoLEM and IndoBERT: A benchmark dataset and pre-trained language model for Indonesian NLP, in: Proceedings of the 28th International Conference on Computational Linguistics (COLING), pp. 757–770.

Li, X., Bing, L., Zhang, W., Lam, W., 2019. Exploiting BERT for end-to-end aspect-based sentiment analysis, in: Proceedings of the 5th Workshop on Noisy User-generated Text (W-NUT), pp. 34–41.

Liu, B., 2012. Sentiment Analysis and Opinion Mining. Synthesis Lectures on Human Language Technologies, Morgan & Claypool Publishers.

Lundberg, S.M., Lee, S.I., 2017. A unified approach to interpreting model predictions, in: Advances in Neural Information Processing Systems (NeurIPS).

Medhat, W., Hassan, A., Korashy, H., 2014. Sentiment analysis algorithms and applications: A survey. Ain Shams Engineering Journal 5, 1093–1113. doi:10.1016/j.asej.2014.04.011.

Mgeni, D.E., 2026. Employing large language models in Swahili, a low-resource language. Natural Language Processing Journal 15, 100209. doi:10.1016/j.nlp.2026.100209.

Nazir, A., Rao, Y., Wu, L., Sun, L., 2022. Issues and challenges of aspect-based sentiment analysis: A comprehensive survey. IEEE Transactions on Affective Computing 13, 845–863. doi:10.1109/TAFFC.2020.2970399.

Ouyang, L., Wu, J., Jiang, X., et al., 2022. Training language models to follow instructions with human feedback, in: Advances in Neural Information Processing Systems (NeurIPS), pp. 27730–27744.

Pontiki, M., Galanis, D., Papageorgiou, H., Androutsopoulos, I., Manandhar, S., AL-Smadi, M., Al-Ayyoub, M., Zhao, Y., Qin, B., et al., 2016. SemEval-2016 task 5: Aspect based sentiment analysis, in: Proceedings of the 10th International Workshop on Semantic Evaluation (SemEval-2016), pp. 19–30.

Pontiki, M., Galanis, D., Papageorgiou, H., Manandhar, S., Androutsopoulos, I., 2015. SemEval-2015 task 12: Aspect based sentiment analysis, in: Proceedings of the 9th International Workshop on Semantic Evaluation (SemEval 2015), pp. 486–495.

Pontiki, M., Galanis, D., Pavlopoulos, J., Papageorgiou, H., Androutsopoulos, I., Manandhar, S., 2014. SemEval-2014 task 4: Aspect based sentiment analysis, in: Proceedings of the 8th International Workshop on Semantic Evaluation (SemEval 2014), pp. 27–35.

Sabty, C., Eyad, M., El Bolock, A., 2026. A comparative study of prompt-based few-shot learning for Arabic and code-switched NLP. Natural Language Processing Journal 15, 100212. doi:10.1016/j.nlp.2026.100212.

Scaria, K., Gupta, H., Goyal, S., Sawant, S.A., Mishra, S., Baral, C., 2024. InstructABSA: Instruction learning for aspect based sentiment analysis, in: Proceedings of NAACL-HLT 2024 (Volume 2: Short Papers), pp. 720–736.

Shidik, G.F., Winarsih, N.A.S., Saraswati, G.W., Saputra, F.O., et al., 2026. A hybrid systematic literature review and automated content analysis for named entity recognition in disaster information management.

Natural Language Processing Journal 15, 100207. doi:10.1016/j.nlp.2026.100207.

Sika, X., Sediyono, E., Sembiring, I., Wahyono, T., Winanto, E.A., 2026. HI-FTT: A hospitality-informed transformer with booking-phase conditioning and semantic group attention for hotel churn prediction. Manuscript under review .

Sun, C., Huang, L., Qiu, X., 2019. Utilizing BERT for aspect-based sentiment analysis via constructing auxiliary sentence, in: Proceedings of NAACL-HLT 2019, pp. 380–385.

Touvron, H., Lavril, T., Izacard, G., et al., 2023. LLaMA: Open and efficient foundation language models. arXiv preprint arXiv:2302.13971 .

Vaswani, A., Shazeer, N., Parmar, N., Uszkoreit, J., Jones, L., Gomez, A.N., Kaiser, L., Polosukhin, I., 2017. Attention is all you need. Advances in Neural Information Processing Systems (NeurIPS) 30.

Wang, Z., Xie, Q., Ding, Z., Feng, Y., Xia, R., 2023. Is ChatGPT a good sentiment analyzer? a preliminary study. arXiv preprint arXiv:2304.04339 .

Wilie, B., Vincentio, K., Winata, G.I., Cahyawijaya, S., Li, X., Lim, Z.Y., Soleman, S., Mahendra, R., Fung, P., et al., 2020. IndoNLU: Benchmark and resources for evaluating indonesian natural language understanding, in: Proceedings of the 1st Conference of the Asia-Pacific Chapter of the ACL (AACL-IJCNLP), pp. 843–857.

Winata, G.I., Aji, A.F., Cahyawijaya, S., et al., 2023. NusaX: Multilingual parallel sentiment dataset for 10 Indonesian local languages, in: Proceedings of EACL 2023, pp. 815–834.

Xue, L., Constant, N., Roberts, A., Kale, M., Al-Rfou, R., Siddhant, A., Barua, A., Raffel, C., 2021. mT5: A massively multilingual pre-trained text-to-text transformer, in: Proceedings of NAACL-HLT 2021, pp. 483–498.

Yan, H., Dai, J., Ji, T., Qiu, X., Zhang, Z., 2021. A unified generative framework for aspect-based sentiment analysis, in: Proceedings of ACL-IJCNLP 2021, pp. 2416–2429.

Zhang, L., Wang, S., Liu, B., 2018. Deep learning for sentiment analysis: A survey. WIREs Data Mining and Knowledge Discovery 8, e1253. doi:10.1002/widm.1253.

Zhang, W., Deng, Y., Liu, B., Pan, S.J., Bing, L., 2024. Sentiment analysis in the era of large language models: A reality check, in: Findings of the Association for Computational Linguistics: NAACL 2024, pp. 3881–3906.

Zhang, W., Li, X., Deng, Y., Bing, L., Lam, W., 2021. Towards generative aspect-based sentiment analysis, in: Proceedings of ACL-IJCNLP 2021, pp. 504–510.

Zhang, W., Li, X., Deng, Y., Bing, L., Lam, W., 2023. A survey on aspect-based sentiment analysis: Tasks, methods, and challenges. IEEE Transactions on Knowledge and Data Engineering 35, 11019–11038. doi:10.1109/TKDE.2022.3230975.

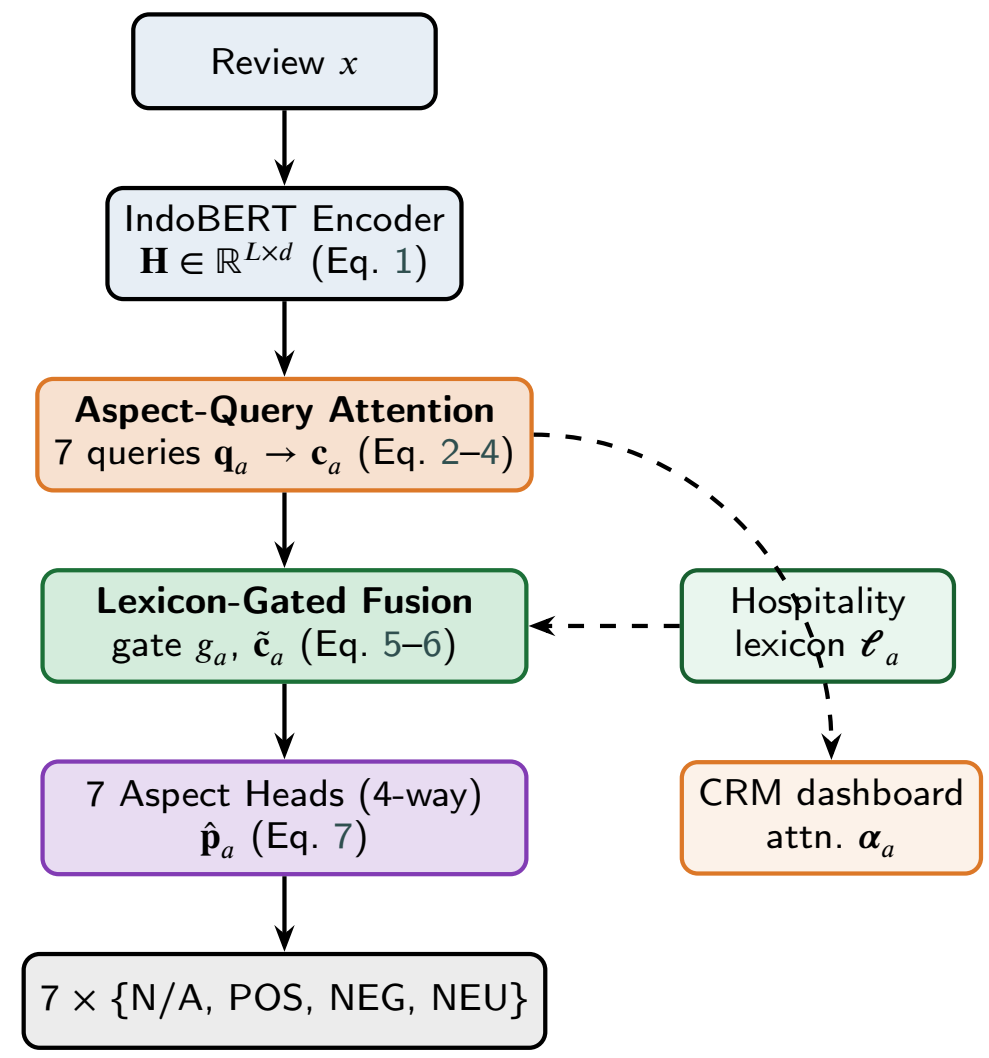

Figure 3: HI-ABSA architecture. An Indonesian encoder feeds Aspect-Query Attention (per-aspect attention pooling), whose weights $\boldsymbol{\alpha}_a$ also drive the CRM dashboard; Lexicon-Gated Fusion injects a hospitality sentiment prior before seven aspect-specific classification heads.

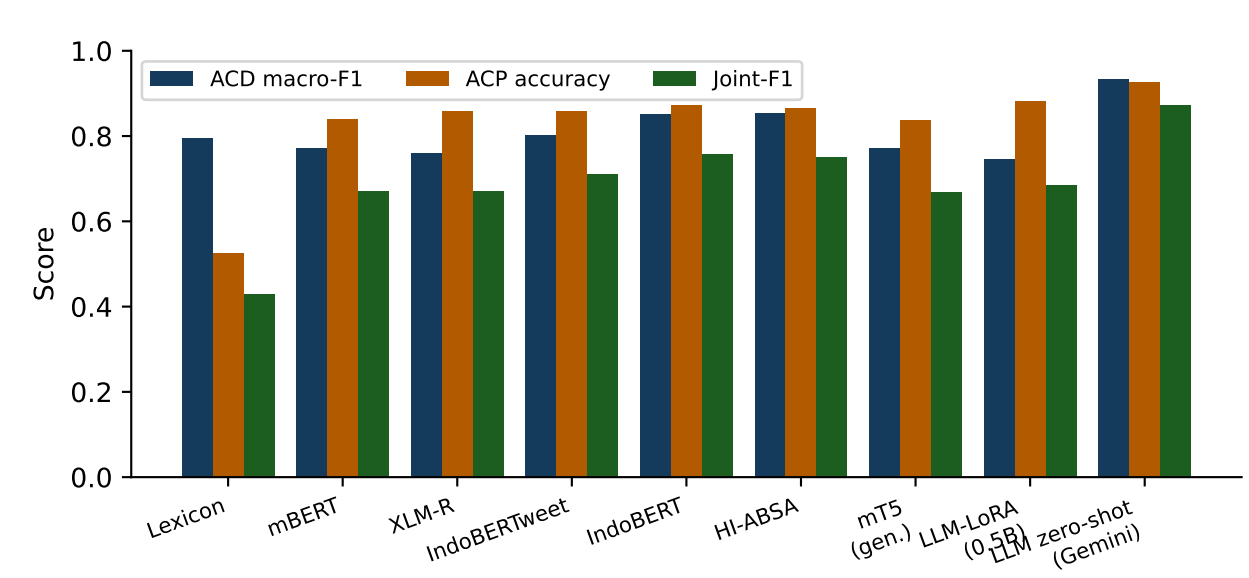

Figure 4: Model comparison on the gold test set across ACD macro-F1, ACP accuracy, and Joint-F1. The zero-shot LLM leads, IndoBERT-based encoders (including HI-ABSA) form a strong middle tier, and both the LoRA-tuned small LLM and the lexicon baseline trail, illustrating that scale and the zero-shot regime, not the LLM paradigm alone, drive the top result.

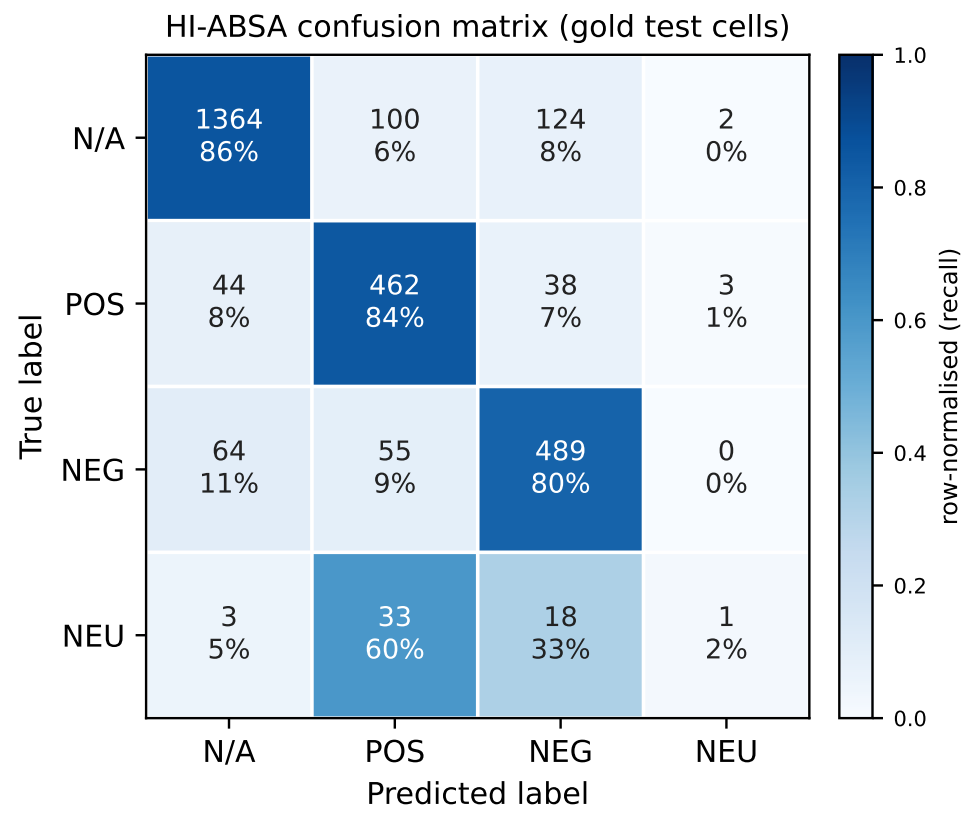

Figure 5: Four-way confusion matrix of HI-ABSA over all (review, aspect) cells of the gold test set (rows: true label; columns: predicted; colour = row-normalised recall, annotated with raw counts). The neutral (NEU) row collapses, almost all true neutrals are predicted positive or negative, while POS/NEG are recovered reliably.

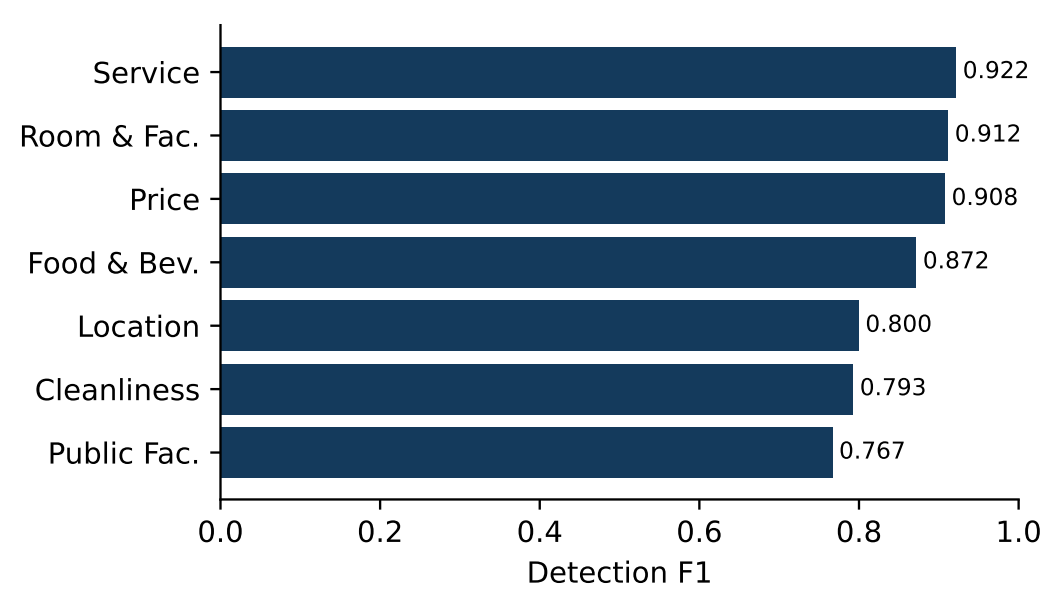

Figure 6: Per-aspect detection (ACD) F1 of HI-ABSA. Explicitly-cued aspects (Service, Room, Price) are easiest; lexically-diffuse and often-implicit aspects (Public Facilities, Cleanliness) are hardest.

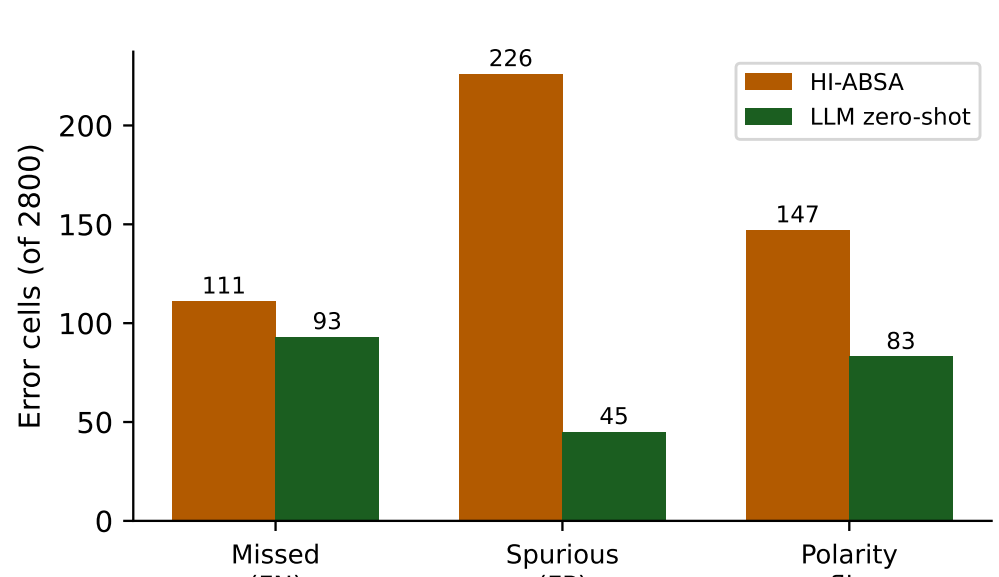

Figure 7: Error-type breakdown on the gold test set. HI-ABSA over-predicts aspects (spurious/false positives dominate), whereas the zero-shot LLM is conservative and errs mainly by missing aspects, consistent with its higher precision.

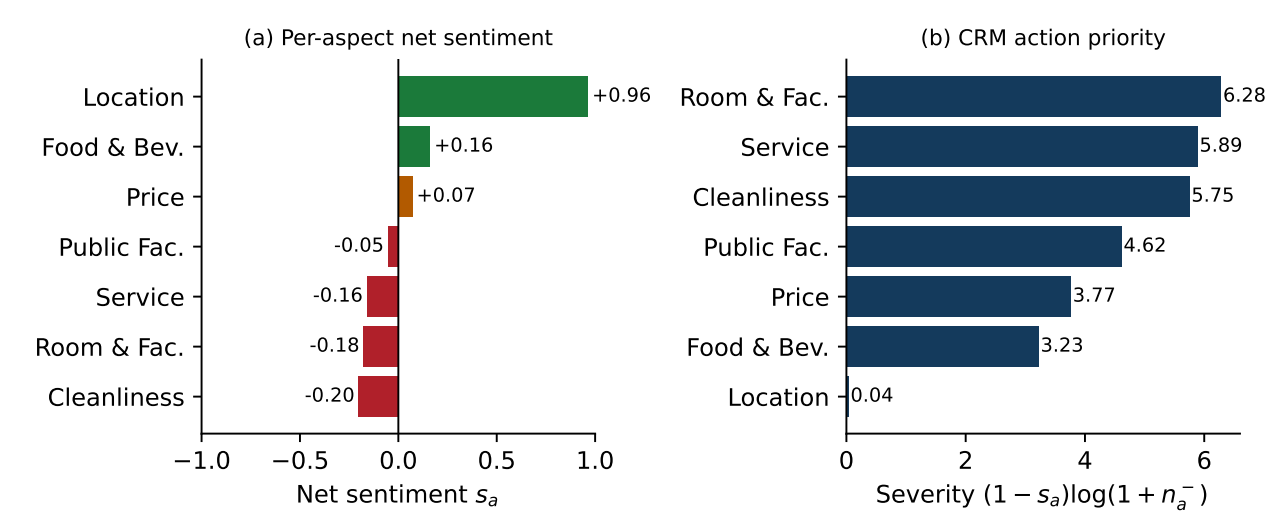

Figure 8: The CRM aspect dashboard instantiated on HI-ABSA predictions (400-review gold test set). (a) Per-aspect net sentiment s_a (Eq. 8): Cleanliness, Room & Facilities, and Service are net-negative; Location is strongly positive. (b) Severity ranking (Section 7.3) prioritising the operational domains that are both negative and frequently mentioned. This aggregate view is the framework's primary, robust interpretability artifact.
